# Customized *MUC1* Sequencing Enables Diagnosis of Autosomal Dominant Tubulointerstitial Kidney Disease and Unexplained CKD

**DOI:** 10.1016/j.ekir.2026.107095

**Published:** 2026-09-25

**Authors:** José María García-Aznar, María Lara Besada-Cerecedo, Nerea López Aguirre, Jorge Pérez Álvarez, Mireia Boluda-Navarro, Sonia González Alvaredo, Iván Lesende, Laura Castañeda-Infante, Lina León-Machado, Alfredo Santana Rodríguez, Vanessa Pérez-Gómez, Elena Gomá Garces, Alberto Ortiz, Gema Fernández Fresnedo, Mercedes Cao Vilarino, Elisabet Ars Criach, Jose Carlos Prado de la Sierra, Rosa María Escaño Marín, Mónica Martín Velázquez, Miquel Blasco, Fabio L. Procaccini, Patricia de Sequera

**Affiliations:** 1 Clinical Area of Nephrology and Immunology, Health in Code, A Coruña, Spain; 2 Genetic Diagnostic Department, Health in Code, A Coruña, Spain; 3 Research and Development Department, Health in Code, Valencia, Spain; 4 Department of Nephrology, Clínica Universitaria de Navarra. Pamplona, Spain; 5 Department of Nephrology, University Hospital La Paz, Madrid, Spain; 6 Clinical Genetics Unit, Complejo Hospitalario Universitario Insular Materno Infantil, Las Palmas de Gran Canaria, Spain; 7 Department of Nephrology, IIS-Fundación Jiménez Díaz University Hospital, Madrid, Spain; 8 School of Medicine, Universidad Autónoma de Madrid, Madrid, Spain; 9 Department of Nephrology, University Hospital Marqués de Valdecilla-IDIVAL, Santander, Spain; 10 Department of Nephrology, University Hospital of A Coruña, A Coruña, Spain; 11 Molecular Genetics Laboratory, Fundació Puigvert, Institut de Recerca Sant Pau (IR-Sant Pau), Barcelona, Spain, ERKNet Reference Center, RICORS2040renal; 12 Department of Nephrology, University Hospital Virgen de la Victoria. Málaga, Spain; 13 Department of Nephrology and Kidney Transplant, National Reference Center for Complex Glomerular Diseases (CSUR), Fundació de Recerca Clínic Barcelona, Institut d'Investigacions Biomèdiques August Pi i Sunyer, Hospital Clínic Barcelona, Barcelona University, Barcelona, Spain; 14 Department of Nephrology, University Hospital Infanta Leonor, Madrid, Spain; 15 University Autónoma de Madrid, Madrid, Spain, and University Complutense de Madrid, Madrid, Spain

**Keywords:** autosomal dominant tubulointerstitial kidney disease, inherited kidney disease, *MUC1*

## Abstract

**Introduction:**

Inherited kidney disease (IKD) is the second most common cause of prevalent kidney failure on kidney replacement therapy in Europe. However, it remains underdiagnosed, in part, because of suboptimal genetic test resolution. The genetic diagnosis of autosomal dominant tubulointerstitial kidney disease (ADTKD) caused by *MUC1* gene variants (ADTKD-*MUC1*) is technically challenging.

**Methods:**

We implemented a customized library for short-read high-throughput sequencing (SR-HTS) with specific bioinformatic approaches to enhance the genetic diagnosis of ADTKD-*MUC1*. Specific analysis of the variable number tandem repeat (VNTR)-*MUC1* region was performed in 550 patients with suspected IKD lacking an identifiable genetic cause using customized SR-HTS. We used a combination of techniques to confirm the genetic variant detected by SR-HTS.

**Results:**

Overall, 28 patients (5% [25/550]) had ADTKD-*MUC1* with DupC (almost 90% [25/28] of *MUC1* variants) or DupA (10%) *MUC1* pathogenic frameshift variants. More than half of these cases required specialized bioinformatics analysis to detect the variants. These variants were validated using Sanger, SNaPshot, or long-read sequencing (LRS) according to the location and mutation type.

**Conclusion:**

This study presents an applicable solution of a customized SR-HTS approach for the systematic screening of *MUC1* gene variants, with particular emphasis on the detection of frameshift alterations within the VNTR region that generate pathogenic *MUC1* neoproteins associated with ADTKD-*MUC1*. Integrating this strategy into routine diagnostic workflows increased the overall diagnostic yield while reducing the technical complexity associated with the sequential application of multiple methodologies.

## Graphical abstract

**Figure:**
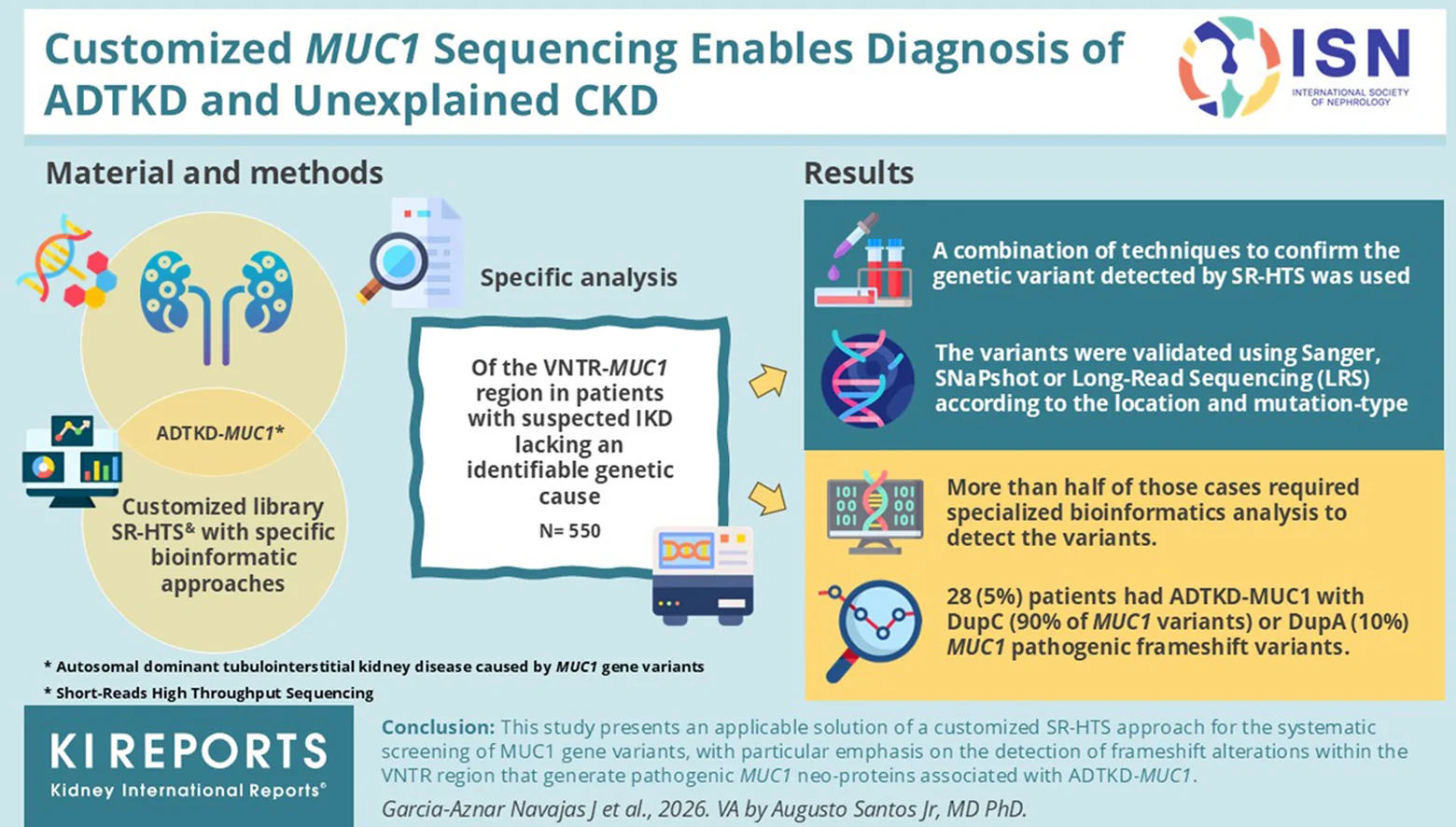

IKD is the second most common cause of prevalent kidney failure on kidney replacement therapy in Europe.^1^ However, it remains underdiagnosed, in part, because of a lack of clinical suspicion or suboptimal access or technical challenges associated with genetic testing. Kidney Disease: Improving Global Outcomes unified the terminology to describe one of the most common IKDs, ADTKD caused by monoallelic variants in the *MUC1, UMOD, REN, HNF1B, or SEC61A1* genes, by adding the name of the causal gene (e.g., ADTKD-*MUC1*). ADTKD shares a common phenotype: progressive loss of kidney function, bland urinary sediment, absent-to-mild albuminuria/proteinuria, no early severe hypertension, tubulointerstitial nephritis not caused by drug exposure, urine concentration defects in children, and normal- or small-sized kidneys. Additionally, each subtype of ADTKD may display specific findings.^1^ ADTKD-*MUC1* may display kidney cysts and intracellular *MUC1* accumulation in distal tubules. Kidney failure occurs between ages 20 and 70 years. Diagnosis requires genetic confirmation of a pathogenic *MUC1* variant.^2^

The presence of pathogenic variants in the *MUC1* gene represents the second most frequent form of ADTKD, explaining approximately 21% of cases.^3^ However, the true prevalence of ADTKD-*MUC1* could not be accurately estimated because described pathogenic variants in *MUC1* are not detectable using conventional next-generation sequencing and standard bioinformatics approaches routinely employed in the genetic diagnosis of other inherited diseases.^4^ *MUC1* variants have traditionally challenged genetic diagnosis because of the intrinsic features of its sequence. *MUC1* encodes Mucin-1, a transmembrane glycoprotein that modulates the permeability of tubular cell surface.^4^ *MUC1* protein contains an extracellular domain with the VNTR region, a transmembrane domain, and a cytoplasmic domain. *MUC1* is proteolytically processed in such a way that it is cleaved into 2 subunits (α/β) through the recognition of a very conserved domain called the SEA module.^5^ The VNTR region consists of modules of 60 repeats that may range from 20 to 125 repeats. The human reference genome version GRCh38/hg38 has recently curated the sequence of *MUC1* (NM_001371720.2), defining 11 exons, with 5 of them (exons 2–6) coding for the VNTR region, in which some differences between modules have been identified. The updated transcript NM_001371720.2 has also been incorporated into the GRCh37/hg19 version.

The most prevalent cause of ADTKD-*MUC1* is a cytosine (C) point duplication in variable repeats in the VNTR region, frequently referred to at chromosomal positions such as NC_000001.10:g.(155160963_155162030)insG for reference isoform NM_001371720.2. This duplication leads to a frameshift producing a neoprotein retained in the endoplasmic reticulum.^6^ The intracellular accumulation of *MUC1*-mutant proteins triggers the unfolded protein response.^7^ The C duplication corresponds to guanine insertion in genomic DNA, as *MUC1* is placed in a negative DNA chain. This variant, classically known as DupC in cDNA, is embedded within a sequence of 7 cytosines, and therefore presents variable C position and variable location of the module. Because of the complex nature of the analysis by high-throughput sequencing (HTS), whole-exome sequencing (WES) can miss this region and cannot be applied to identify any variant in the VNTR region reliably enough for genetic diagnosis. In contrast, some manufacturers include products with enrichment of WES or customized libraries of sequencing that specifically cover the VNTR region, allowing capture of genetic alterations, but need a proper bioinformatic pipeline to suitably analyze this region. To solve this challenging region, SNaPshot was widely used as the standard method for VNTR-*MUC1* assays, targeted to the commonly known c.428dupC.^8^ SNaPshot allows detecting the presence/absence of this specific variant but cannot inform about the location among the different VNTR-*MUC1* modules and does not identify different genetic variations with functional effects similar to those of DupC.^9^

Other approaches have been proposed to improve VNTR-*MUC1* region analysis. Specific amplification of the VNTR-*MUC1* region by long-range PCR was proposed to prepare Single-Molecule Real-Time Sequencing libraries sequenced by Pacific Biosciences (Menlo Park, CA) and later assembled to a genome reference.^10^ This procedure enabled the accurate identification of cytosine duplications within the VNTR-*MUC1* region in patients diagnosed with ADTKD. To reduce the limitations of short-read (SR) sequencers, LRS was introduced to improve the yield of HTS in a customized library for VNTR*-MUC1* with comparable results to SNaPshot in Japanese patients having ADTKD.^11^ Very recently, a standardized computational pipeline, called VNTyper, was validated to genotype VNTR-*MUC1* regions using SR sequencing, which showed the clinical validity and utility of HTS to replace non-HTS techniques.^12^ Other pipelines have been developed with high sensitivity and specificity to detect the DupC variant, such as SharkVNTyper, although no other frameshift variants in the VNTR region have been detected in the large cohort of patients evaluated with this approach.^13^ Single-Molecule Real-Time Sequencing for DupC and other *MUC1*-frameshift variants was extensively validated and compared with a standardized probe-extension assay, obtaining 100% concordance at the family level and 2% discordance at the individual level, likely attributable to allelic dropout during LRS.^14^

We have now implemented a customized library by SR-HTS in conjunction with specific bioinformatic approaches to enhance the genetic diagnosis of ADTKD-*MUC1*. This approach enables comprehensive characterization of the mutational landscape while streamlining diagnostic workflows by reducing the need for multiple, technique-specific assays for a single suspected disorder.

## Methods

### VNTR Region

Genome reference GRCh37/hg19 proposed that *MUC1* was encoded by 8 exons, defining the VNTR region between exons 2 and 3 and encompassing a region of over 700 base pairs with high homology corresponding to the tandem repeats of 60 nucleotides.

### Patient Cohort

During 2020–2025, we sequenced patients with unexplained CKD (CKDx) using SR-HTS and an enriched library prepared with the SureSelect HS protocol (Agilent Technologies, Santa Clara, CA, USA) that included 529 genes associated with IKD. In some patients, CKD was the only available clinical information; therefore we could not subcategorize all patients into different phenotypes. Specific analysis of the VNTR-*MUC1* region for cytosine or thymine duplications was performed in 550 patients in whom we did not identify a genetic cause, that is, we had not found pathogenic, likely pathogenic, or uncertain significance variants with high suspicion, classified according to the American College of Medical Genetics and Genomics.^15^^,^^16^

### DNA Short-Read Sequencing and Analysis

A customized HTS library for target-gene capture of 529 genes was used as previously described.^17^ DNA was extracted from peripheral blood or saliva samples using automated genomic DNA purification (QIAsymphony SP, Qiagen, Venlo, the Netherlands). Samples were prepared using the Agilent SureSelect (Santa Clara, CA) Library Preparation Kit for Illumina paired-end multiplexed sequencing according to the manufacturer’s instructions. We enriched regions of interest with probes to adequately cover all coding exons and 50 base pairs of flanking intronic sequences and the whole VNTR region of *MUC1*. Cluster preparation was performed using the cBot (Illumina, San Diego, CA, USA) device.

Bioinformatics analysis was performed in accordance with the best WES analysis practices based on the recommendations from the GATK Broad Institute and GATK4 versions (https://gatk.broadinstitute.org/*).* Sequencing data were analyzed using a proprietary bioinformatics pipeline that includes both sample demultiplexing (using Illumina software) and the required steps to obtain an annotated variant report, along with the corresponding coverage and quality parameters. The pipeline follows this logical sequence: alignment; alignment refinement and adjustment; variant calling; variant normalization and quality scoring; and generation of coverage statistics by region of interest and copy number variation detection using an in-house statistical method based on depth of coverage. This test can identify single-nucleotide variants and insertions/deletions of up to 50 base pairs. Genetic variants in the *MUC1* gene were aligned using the reference genome GRCh37/hg19 and reported following Human Genome Variation Society recommendations (www.hgvs.org). This procedure was applied in the non-VNTR region of the *MUC1* gene as well as the first 2 repeats of this region, which contain a specific sequence whose alignment may be mapped to the reference human genome.

### Bioinformatics Analysis for the VNTR-*MUC1* Region

A specific computational algorithm of filtering was applied to select reliable relevant variants in the VNTR-*MUC1* region. Conventional WES pipelines, as noted above, were not able to specifically align the repeats of the VNTR region, generating numerous ambiguities and low quality reads; therefore, variant callers could not detect the variants located in deep modules of this region. The first step considered variants with no restriction on alternative allele frequency and parameters of quality for variant calling. The mean depth of read of this region was >14,000x. We focused on relevant described changes, those single-nucleotide variants/insertions or deletions predicted to produce frameshift and nonsense variants within the VNTR region (Supplementary Table S1).

A custom bioinformatic short-read analysis workflow was developed to evaluate candidate repeat-containing fragments in the *MUC1*-VNTR region from aligned BAM files (Supplementary material S2).

We specifically searched for the most prevalent variant in the *MUC1* gene, through the identification of 8 guanines (reverse complement chain) in the wild-type sequence of CTGGGGGGGCGG by using an in-house bioinformatic pipeline based on Samtools.^18^ We calculated the ratio of mutant reads with an extra guanine (or cytosine in the reverse chain) compared with all reads in the different modules of the VNTR. We also investigated other mutation types, such as deletions and insertions, whose frequency among total reads increased compared with the normal ratio in the same way as with 8 guanines. In this instance, the second most recurrent variant was DupA (CTTGGGGGGGCGG), which was also tested in this study.

We further searched for other reported variants in this region such as the variants registered in the dictionary of *MUC1* variants: https://github.com/pristanna/muc1repeats.

### Sanger Sequencing

We optimized Sanger sequencing to amplify and sequence VNTR-MUC1 repeated modules. We used a GC-rich protocol for the PCR system and the following PCR primers, especially designed for the VNTR region: Forward 1: GGAGAAAAGGAGACTTCGGCTACCCAG and Reverse: TCCGGCCTGCTCTCCGAGGCCGA. Then, we sequenced the PCR amplicon by using 2 forward primers and 1 reverse primer because of the high noise of signal in the reverse sequencing: Forward 1: GGAGAAAAGGAGACTTCGGCTACCCAG, Forward 2: ACAGGATGTCACTCTGGC, and Reverse: TCCGGCCTGCTCTCCGAGGCCGA.

### SNaPshot

The presence or absence of cytosine duplication in the VNTR region of *MUC1* [c.428dupC] was screened in 12 samples by using SNaPshot (Thermo Fisher Scientific, Waltham, MA) minisequencing, as previously described.^8^

### DNA Long-Read Sequencing and Analysis

LRS was performed on the Pacific Biosciences platform using a WGS library approach. gDNA quality was assessed using the Femto Pulse gDNA 165 kb assay. Low-molecular-weight fragments (<10 kb) were removed using the Short-Read Eliminator kit, and the resulting gDNA was subsequently sheared to obtain fragments larger than 10 kb. Libraries were prepared using PacBio SMRTbell prep kit 3.0 (Menlo Park, CA) according to the manufacturer’s instructions. High-fidelity reads were generated by sequencing on the Revio system (PacBio). Bioinformatic analysis was performed using the PacBio High-fidelity human WDL-WGS pipeline (v1.2.0), including read alignment with pbmm2.

A custom bioinformatic workflow was developed to identify and characterize repeat motifs in the *MUC1*-VNTR region from aligned long-read sequencing data (Supplementary material S3).

### Statistical Analysis

We used a statistical model based on data from the number of reads carrying the mutant allele and read depth from 550 samples to validate the threshold of positive samples for *MUC1-*DupC and the critical range for decision-making. We established 2 sample groups: confirmed results (positive zone—high probability of confirmed variant) and unconfirmed results (negative zone—likely technical noise or unconfirmed variant). To ensure the robustness of the model, a Mann-Whitney U test compared the frequency distributions between confirmed and unconfirmed groups. A linear regression model (*R*^2^ > 0.95) was applied to validate sample quality across expected mutated reads according to 4 scales of read depth in the VNTR region (0–5000x; 5001–10,000x; 10,001–20,000x; 20,001–30,000x).

### Informed Consent

All patients provided signed informed consents for genetic analysis. This work did not include clinical data, and all individuals were anonymized with regard to genetic results.

## Results

### VNTR-MUC1 mapping

Alignment analysis of the VNTR-*MUC1* region revealed high mappability in the flanking sequences, including the first, second, and terminal repeat modules within the VNTR. Visualization using the Integrative Genomics Viewer confirmed that both forward and reverse reads from BAM files were specifically mapped to these regions.^19^ However, within the central VNTR modules, read mapping lacked specificity because the alignment algorithm was unable to unambiguously localize sequences to individual tandem repeats (Figure 1). This limitation arises from the high interindividual variability of VNTR repeats and the absence of a consensus human reference sequence for this locus. Consequently, standard WES approaches failed to achieve complete coverage of the VNTR*-MUC1* region. To address this, we performed targeted screening for the characteristic C-duplication within the VNTR (corresponding to a guanine insertion at the chromosomal level) using a customized SR-HTS, which enabled full coverage of the VNTR*-MUC1* region (Figure 1).

**Figure 1:**
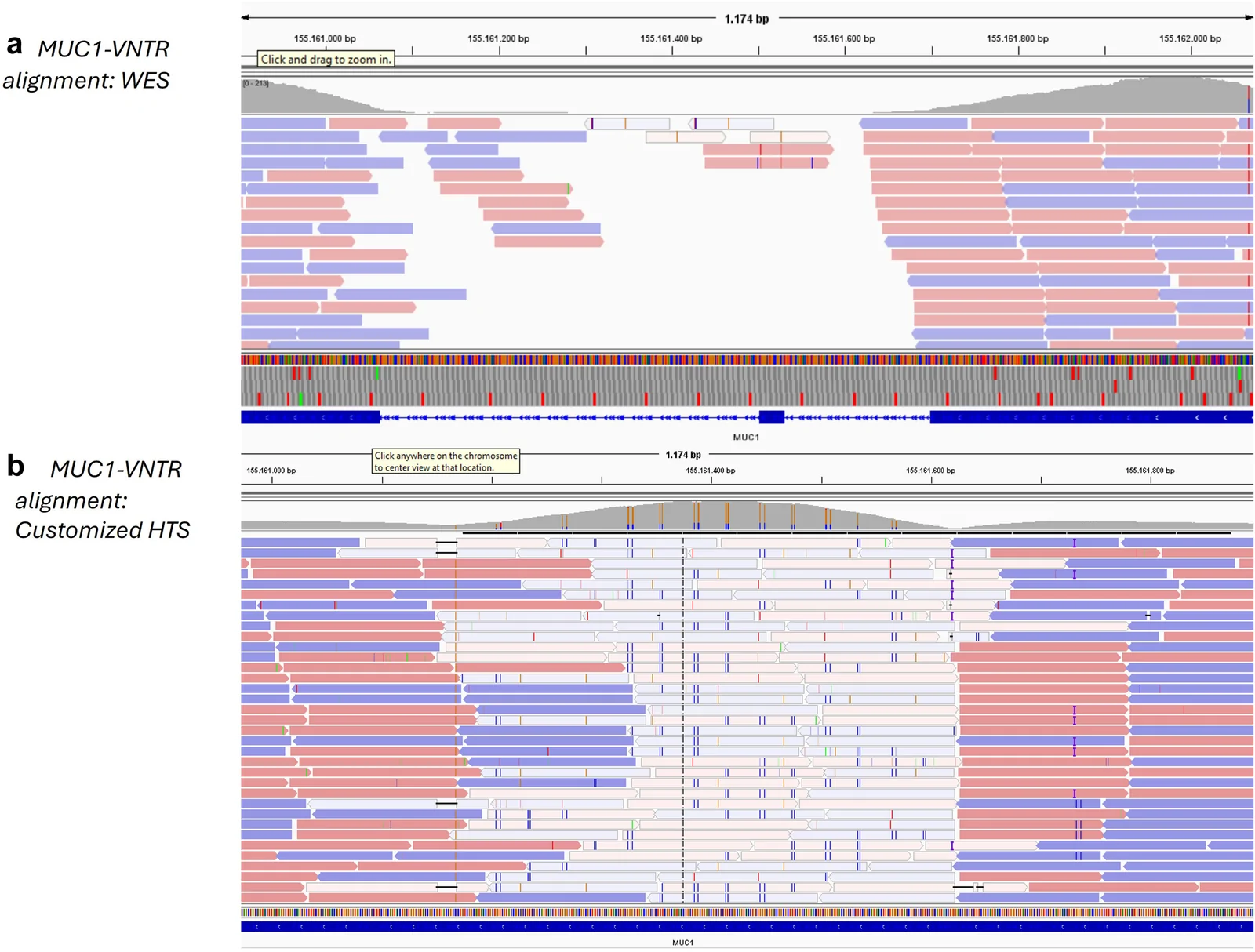
Comparative coverage of the *MUC1-*VNTR region between WES and the customized HTS panel. (a) Read alignment from WES mapped against the VNTR region. (b) Read alignment from customized NGS mapped against the VNTR region. NGS, next-generation sequencing.

Additionally, we screened for other potential genetic variants within the VNTR region that could introduce premature stop codons from the dictionary of *MUC1* variants (https://github.com/pristanna/muc1repeats/blob/main/repeat_list.md), potentially leading to functional consequences similar to those caused by the DupC variant. From this list, we only detected the DupA variant.

### Cohort Analysis for DupC Variant

We analyzed 550 samples without a genetic cause according to SR-HTS, which achieved full coverage of the VNTR*-MUC1* region. Pathogenic DupC within the VNTR was identified in 25 patients. A total of 12 patients were heterozygous for the previously reported variant p.Ala144Serfs∗86, located in the first VNTR repeat, and 1 carried the p.Ala164Serfs∗106 variant in the second repeat. The DupC variant in the first 2 VNTR repeats was detected by conventional variant calling with an allele frequency ranging from 32% to 47%, consistent with the expected profile of a heterozygous variant. This frequency falls within the range typically observed for germline variants in well-mapped genomic regions. Notably, the first and second VNTR repeats exhibited high mappability because of their proximity to the exon start, a region with near-complete mapping efficiency in the *MUC1* gene. All samples harboring duplications in these repeats were subsequently validated by bidirectional Sanger sequencing (Figure 2). Samples with DupC in internal modules different from the first and second repeats could not be detected by conventional variant calling; therefore, they were analyzed using a specialized approach.

**Figure 2:**
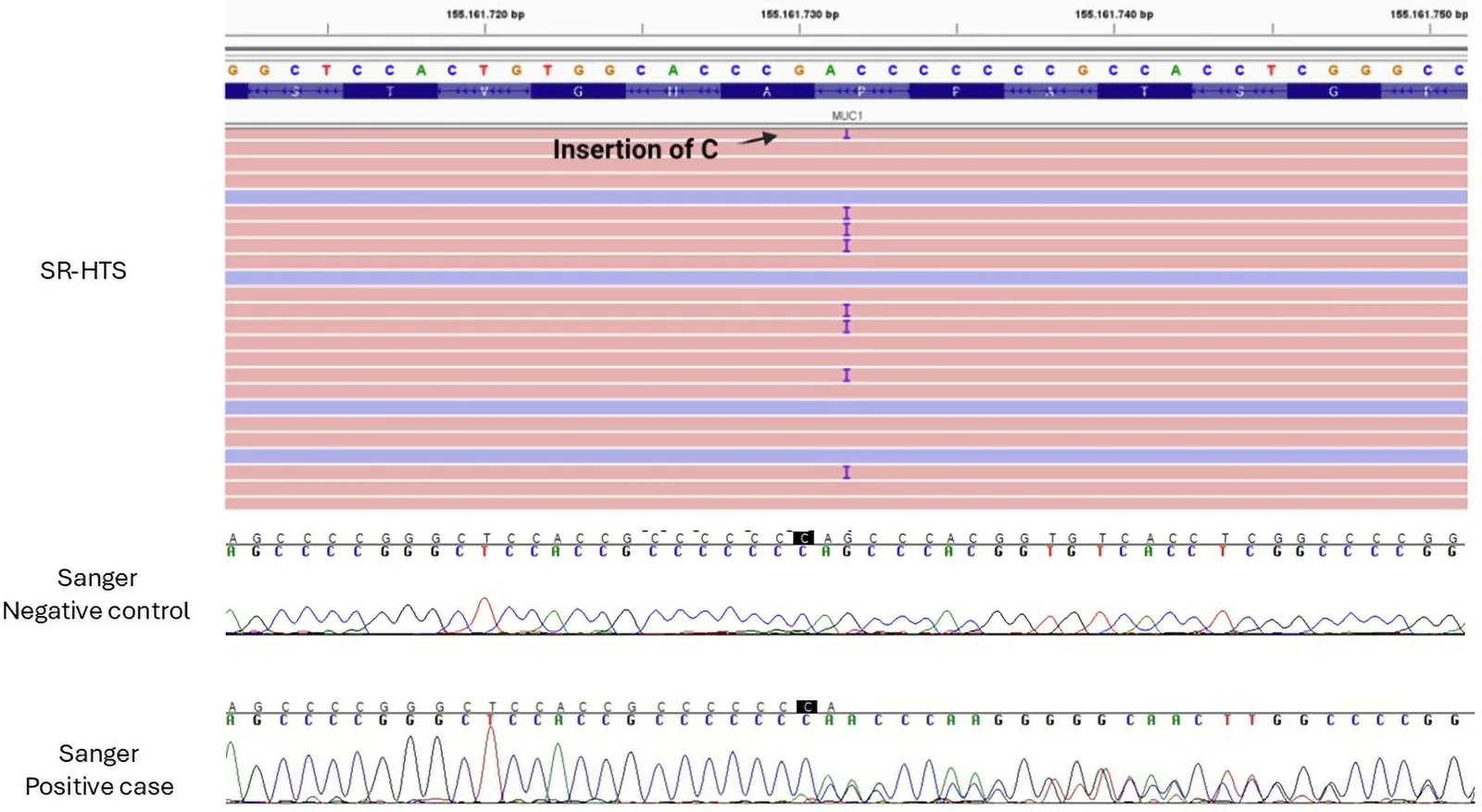
Visualization of the c.428dup (DupC) variant using the Integrative Genomics Viewer (IGV and Sanger sequencing electropherograms. The IGV alignments enable reliable detection of the DupC variant when located within the first VNTR module, owing to the high mappability of this region. The variant is also identified by the variant-calling pipeline with an alternative allele frequency within the expected range for a heterozygous variant. The presence of the DupC variant was confirmed by bidirectional Sanger sequencing, as illustrated in the electropherogram, where a characteristic double peak is observed at the insertion site.

When the C duplication occurred within the internal VNTR modules, it was embedded in highly homologous sequences repeated up to 125 times per allele, as previously reported. Despite using a consistent sequencing protocol, we observed substantial variability in read depth, ranging from 1000× to more than 45,000×. This variability likely reflects significant heterogeneity in VNTR length among alleles within this cohort. Given that an individual may carry 2 alleles with up to 125 repeats in the VNTR region of *MUC1*, a single C duplication or any point variant within this region would be expected to yield a theoretical allele frequency of >0.4%. This low expected frequency necessitates the use of highly sensitive detection methods capable of reliably identifying low-frequency variants instead of standard conventional variant calling.

To address this limitation, we specifically searched for sequences containing 8 consecutive guanines (or cytosines on the reverse strand) within the motif CT(G)GGGGGGCGG across the 550 samples. This strategy enabled estimation of the proportion of mutant reads carrying an additional guanine relative to wild-type reads.

Most individuals exhibited fewer than 50 reads containing 8 consecutive guanines, corresponding to a ratio ≤0.4% of total read depth. In contrast, 12 patients showed evidence of a guanine within the CTGGGGGGGCGG sequence, with allele frequencies ≥0.6% and more than 50 supporting reads in at least 1 VNTR module (Figure 3). One exception was observed in a patient with a DupC ratio of 0.54%, supported by 28 reads out of 5197 total reads.

**Figure 3:**
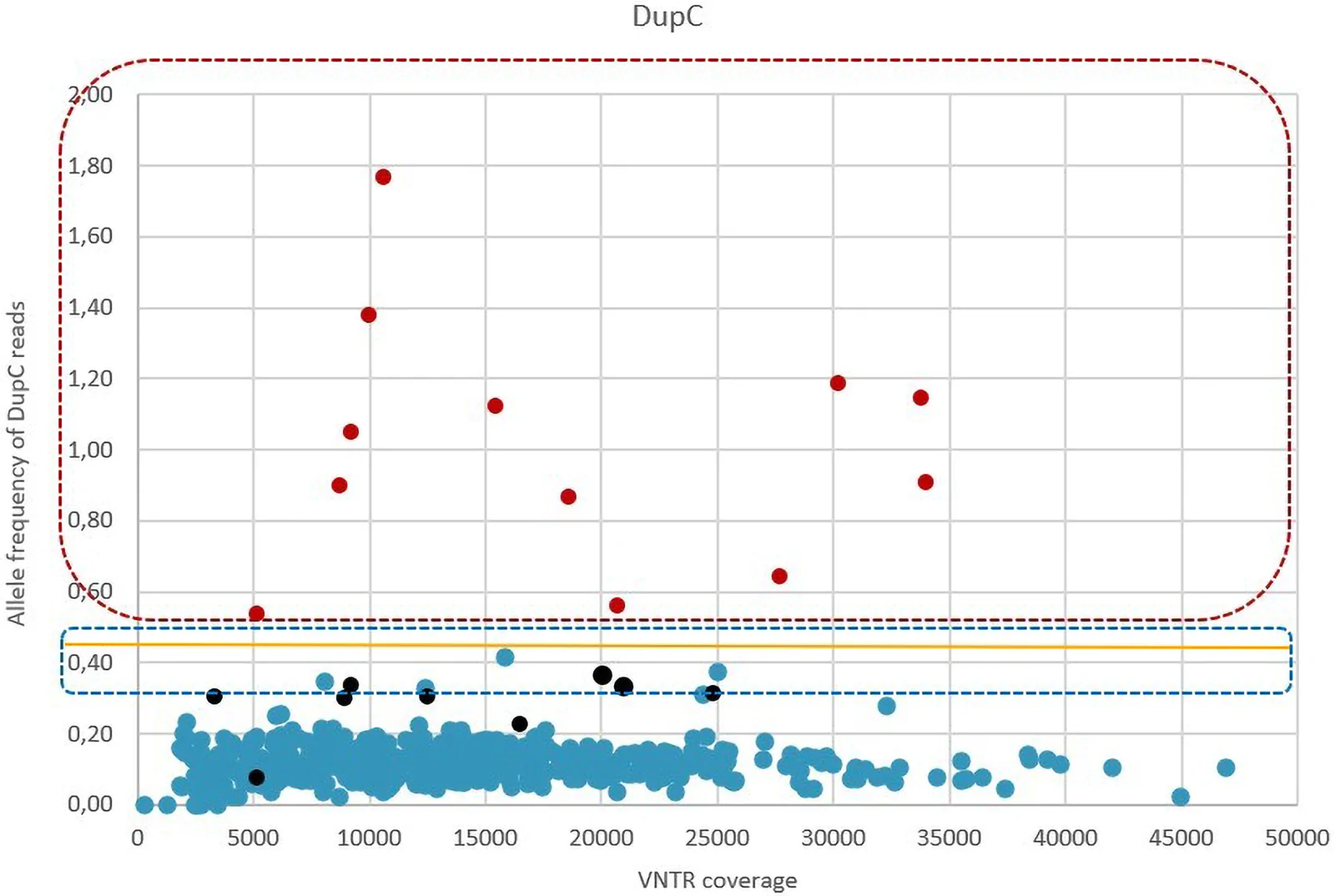
Graphical representation of the relationship between mutant allele read frequency and sequencing depth among 550 patients for the NC_000001.10:g.(155160963_155162030)insG variant. Red circles represent true-positive cases for the DupC variant. Black circles indicate true-negative cases. Blue circles represent negative cases without secondary confirmation, observed in most individuals. The red dashed line denotes the positive zone with high sensitivity (ratio ≥ 0.5), whereas the blue dashed lines define the zone of uncertainty (ratio 0.36–0.54). The orange line depicts the threshold (0.45) of variant frequency to consider a potential positive variant, which should be confirmed.

To confirm the presence of DupC variants, we performed bidirectional Sanger sequencing for cases in which the duplication was located within the first 2 VNTR repeats, as these were reliably detected by next-generation sequencing variant-calling pipelines. Samples showing evidence of C duplications that were not identified by standard variant callers—suggesting localization within internal VNTR modules—were further analyzed using the SNaPshot assay or long-read sequencing on the Pacific Biosciences platform.

Overall, 25 patients were identified as carriers of a pathogenic DupC variant within the VNTR-*MUC1* region. These findings were validated using 3 complementary techniques, selected according to the inferred location of the repeat module (Sanger sequencing, SNaPshot, and LRS; Table 1).

**Table 1:** List of *MUC1*-DupC variants detected in this study

Sample	Variant position	Alternative allele frequency (%)	Confirmation
M-1	NM_001371720.2: c.428dup; p.Ala144Serfs∗86	40%	Sanger
M-2	NM_001371720.2: c.428dup; p.Ala144Serfs∗86	46%	Sanger
M-3	NM_001371720.2: c.428dup; p.Ala144Serfs∗86	38%	Sanger
M-4	NM_001371720.2: c.428dup; p.Ala144Serfs∗86	41%	Sanger
M-5	NM_001371720.2: c.428dup; p.Ala144Serfs∗86	45%	Sanger
M-6	NM_001371720.2: c.488dup; p.Ala164Serfs∗106	47%	Sanger
M-7	NM_001371720.2: c.428dup; p.Ala144Serfs∗86	40%	Sanger
M-8	NM_001371720.2: c.428dup; p.Ala144Serfs∗86	44%	Sanger
M-9	NM_001371720.2: c.428dup; p.Ala144Serfs∗86	42%	Sanger
M-10	NM_001371720.2: c.428dup; p.Ala144Serfs∗86	45%	Sanger
M-11	NM_001371720.2: c.428dup; p.Ala144Serfs∗86	32%	Sanger
M-12	NM_001371720.2: c.428dup; p.Ala144Serfs∗86	40%	Sanger
M-13	NM_001371720.2: c.428dup; p.Ala144Serfs∗86	34%	Sanger
M-14	NC_000001.10:g.(155160963_155162030)insG	0.91%	SNaPshot
M-15	NC_000001.10:g.(155160963_155162030)insG	1.76%	LRS
M-16	NC_000001.10:g.(155160963_155162030)insG	1.12%	SNaPshot
M-17	NC_000001.10:g.(155160963_155162030)insG	1.38%	SNaPshot
M-18	NC_000001.10:g.(155160963_155162030)insG	0.56%	LRS
M-19	NC_000001.10:g.(155160963_155162030)insG	1.05%	LRS
M-20	NC_000001.10:g.(155160963_155162030)insG	0.90%	SNaPshot
M-21	NC_000001.10:g.(155160963_155162030)insG	0.64%	LRS
M-22	NC_000001.10:g.(155160963_155162030)insG	0.87%	SNaPshot
M-23	NC_000001.10:g.(155160963_155162030)insG	1.18%	SNaPshot
M-24	NC_000001.10:g.(155160963_155162030)insG	1.15%	SNaPshot
M-25	NC_000001.10:g.(155160963_155162030)insG	0.54%	SNaPshot

LRS, long-read sequencing; NM, NCBI mRNA reference sequence; NC, NCBI genomic reference sequence; VNTR, variable number tandem repeat.

The variants shown in this table were referred to the genome reference GRCh37 hg19 and isoform NM_001371720.2. We added information on the alternative allele frequency of the reads containing the mutation and the alternative technique used for confirmation of the variant. The variant c.428dup was located in the first module of the VNTR region, and the variant c.488dupC was located in the second module.

Four samples testing positive for the DupC variant were subsequently confirmed by LRS. To enable accurate analysis, we developed a custom alignment tool specifically designed to visualize the VNTR region of the *MUC1* gene. This approach allowed precise detection of the DupC variant and its exact localization within the VNTR, overcoming the limitations of other methods such as SNaPshot and SR sequencing, which do not provide positional resolution within repetitive regions. This method enabled determination of the number of repeats in each allele and, consequently, the precise position of DupC within the VNTR region (Figure 4).

**Figure 4:**
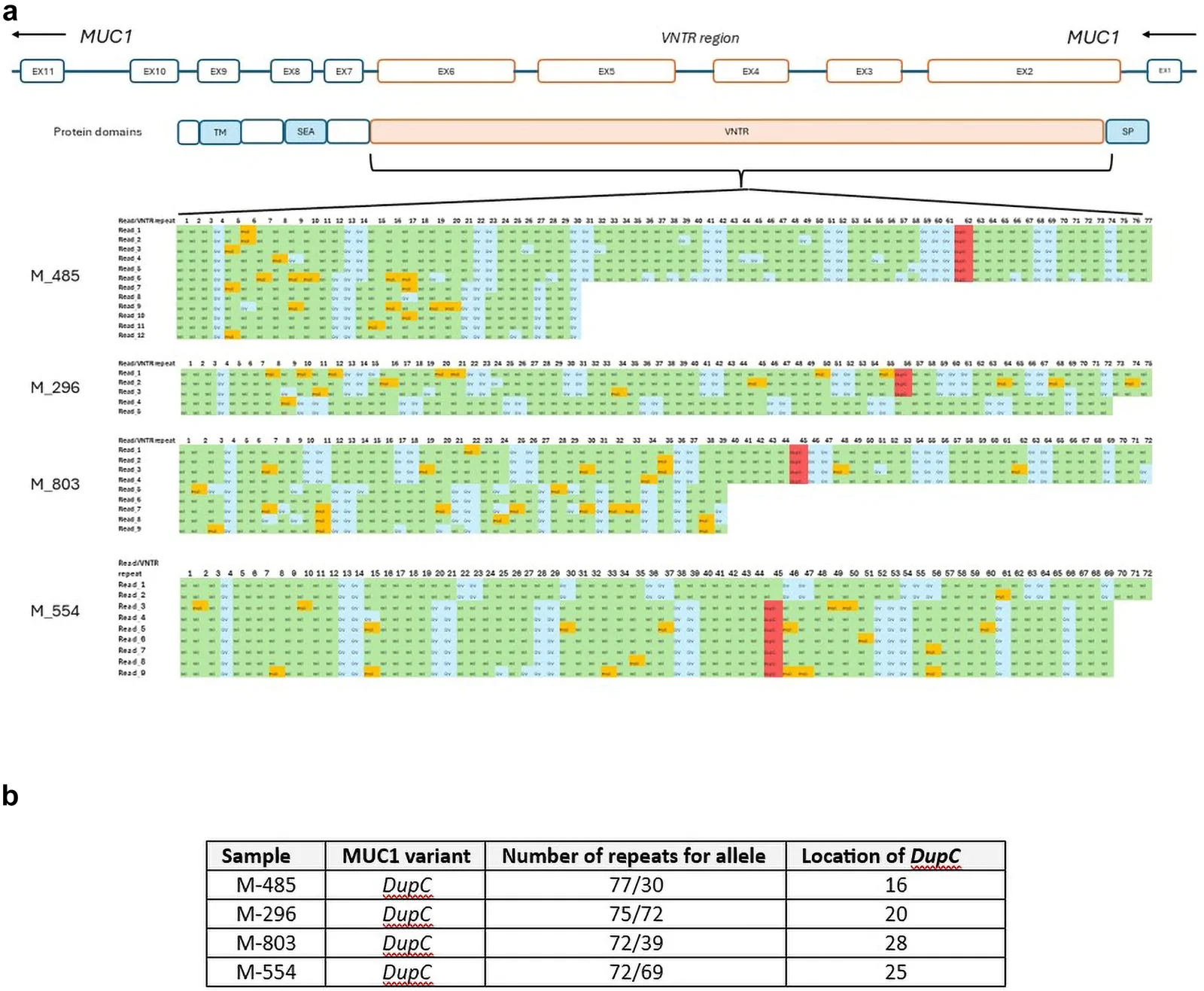
Phasing analysis and alignment of LRS in 4 samples positive for the DupC variant in the *MUC1* gene. (a) Graphical representation of the number of reads containing VNTR modules, distinguishing both alleles. Wild-type modules are represented in green; slight differences (< 90%) from the consensus module are represented in blue; *MUC1*-frameshift variants are represented in orange; and the target mutation (DupC) is represented in red. DupC is aligned in the same repeats on the affected allele. (b) Number of repeats and location for each sample.

### Validation of DupC Detection by SR-HTS

The diagnostic performance of the detection model for the identification of DupC variants within internal repeats of the VNTR-*MUC1* region was validated using a control cohort (n = 21) with previously confirmed results. These samples included the 12 true-positive samples for DupC and 9 true-negative samples.

The model correctly identified all positive cases (12/12), with variant allele frequencies ranging from 0.54% to 1.76%, and all negative cases (9/9), which showed frequencies between 0.14% and 0.36%, with no false-positive results observed. Five samples with a frequency >0.30% and <0.54% were not tested by orthogonal methods; therefore, these were considered a zone of uncertainty for DupC. These included patients with a variant frequency of 0.30% (M-108), 0.31% (M-184), 0.38% (M-79), 0.38% (M-149), and 0.42% (M-156). Based on these findings, an optimal detection threshold was established at an alternative allele frequency cutoff of 0.45%. This score defined the positive signal to confirm the genetic defect. The positive zone (>0.45%) indicated a high probability of a confirmed variant, and the negative zone (<0.45%) indicated likely technical noise or an unconfirmed variant.

The Mann-Whitney U test determined 2 groups without overlapping minimum true-positive value and maximum true-negative values, confirming that the results were not because of chance. The difference between groups was statistically highly significant (at a 95% confidence level, α = 0.05). The absence of overlap between the ranges allowed us to select a threshold for decision-making.

### DupA Frameshift Variants in the VNTR-*MUC1* Region

SR-HTS in the 550 samples also identified 3 patients carrying a previously described thymine duplication (DupA) immediately preceding a homopolymeric stretch of 7 guanines: CT(T)GGGGGGGCGG.^9^ This variant was annotated as NC_000001.10:g.(155160963_155162030)insT, analogous to the annotation used for DupC. All 3 patients presented with a clinical diagnosis of tubulointerstitial nephropathy.

In a clustering analysis for DupA, performed using the same strategy applied for DupC detection, these 3 patients had an allele frequency between 0.7% and 1.4%, whereas all other samples were ≤ 0.1% (Figure 5). DupA was supported by 249, 197, and 60 reads, respectively, with a total read depth ranging from 8000× to 29,000×. Alignment visualization using Integrative Genomics Viewer excluded localization of DupA within the first 2 VNTR repeats, suggesting localization within internal repeat modules.

**Figure 5:**
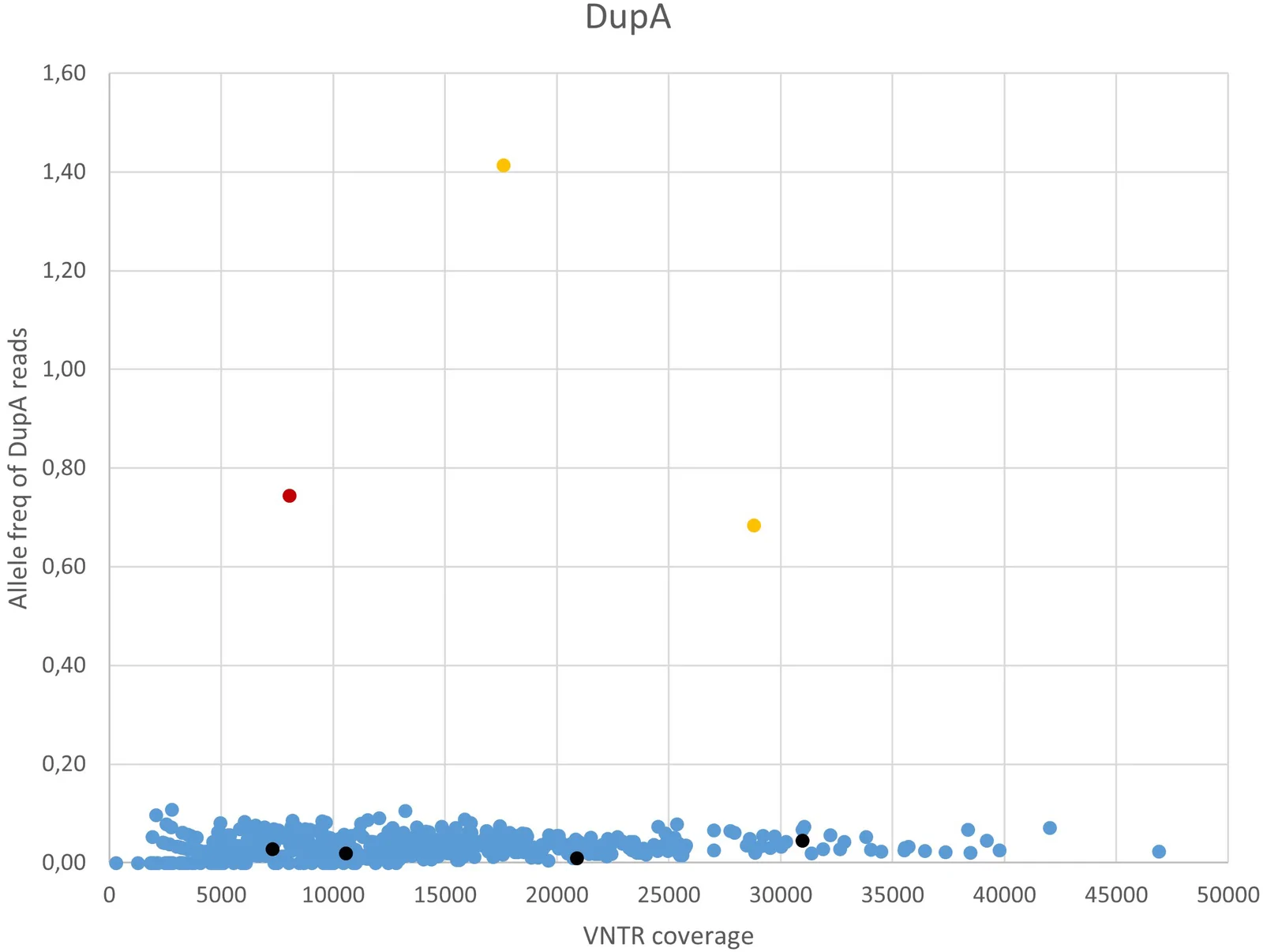
Graphical representation of the relationship between mutant allele read frequency and sequencing depth among 550 patients for the NC_000001.10:g.(155160963_155162030)insT variant. Red circles represent true-positive cases for the DupA variant. Black circles indicate true-negative cases. Blue circles represent negative cases without secondary confirmation, observed in most individuals. Orange circles represent patients from the same family.

The DupA variant could not be validated using the SNaPshot assay, because this method was specifically designed to target DupC. LRS could not be performed because of limited quality of the sample for optimal LRS. However, in one case with a strong family history of kidney failure on kidney replacement therapy, the DupA variant was detected in a relative with ADTKD at an allele frequency of 0.7% using SR-HTS and was subsequently confirmed by LRS (Table 2). These findings support the presence of the DupA variant and its segregation with the familial disease phenotype. The other patient who was potentially positive for DupA harbored a higher ratio for the DupA variant, but secondary testing was not performed.

**Table 2:** List of *MUC1*-DupA variants detected in this study

Sample	Variant position	Alternative allele frequency (%)	Confirmation
M-34∗	NC_000001.10:g.(155160963_155162030)insT	1.41%	Not performed
M-550	NC_000001.10:g.(155160963_155162030)insT	0.68%	Not performed
M-31∗	NC_000001.10:g.(155160963_155162030)insT	0.74%	LRS

LRS*,* long-read sequencing; NC*,* NCBI genomic reference sequence.

Variants are referred to genome reference GRCh37 hg19 and isoform NM_001371720.2. ∗Patients from the same family.

Clustering analysis to establish a cutoff by this method was not performed because of the low number of positive cases for DupA and the limitation of true positives from the lack of a secondary confirmatory test. However, graphical analysis showed a negative zone with a score <0.2 consistent with data for true-negative cases.

We did not perform a benchmarking of our approach with VNTyper. Nevertheless, we submitted BAM files of positive samples from our validation https://vntyper.org.^10^ From the 12 true-positive samples for DupC located in deep modules, 10 samples (83.3%) were detected by VNTyper with high precision, 1 sample (8.3%) was detected by VNTyper with low precision, and 1 sample (8.3%) was not detected by this platform. From the 3 true-positive samples for DupA, only 1 patient (33.3%) was positively detected by VNTyper.

Overall, we genetically resolved 28 (5%) patients affected by ADTKD for DupC or DupA *MUC1* variants among patients with suspected IKD but negative on initial genetic testing using a standard pipeline, thereby expanding the spectrum of pathogenic variants classically reported in the *MUC1* gene. Of these, 90% harbored the most prevalent pathogenic variant, DupC, whereas 10% carried the alternative frameshift DupA variant within the same VNTR region (Table 3). The summary of clinical information for the 28 positive patients is shown in Table 4. In 28.6% of the patients (8/28) ADTKD was suspected or diagnosed, whereas 71.4% (20/28) had unexplained chronic kidney disease with or without a family history of nephropathy.

**Table 3:** List of frameshift variants identified in the VNTR-*MUC1* region for this cohort

*MUC1* variant (cDNA and protein change)	No. of CKD patients	Mutation type	Location	ACMG classification	Confirmation by orthogonal technique
NM_001371720.2: c.428dup; p.Ala144Serfs∗86	12	Frameshift	VNTR (module 1)	Pathogenic (PVS1, PM2, PS3, PS4, PP5)	Yes
NM_001371720.2: c.488dup; p.Ala164Serfs∗106	1	Frameshift	VNTR (module 2)	Pathogenic (PVS1, PM2, PS3, PS4, PP5)	Yes
NC_000001.10:g.(155160963_155162030)insG	12	Frameshift	VNTR (deep module)	Pathogenic (PVS1, PM2, PS3, PS4, PP5)	Yes
NC_000001.10:g.(155160963_155162030)insT	3	Frameshift	VNTR (deep module)	Pathogenic (PVS1, PM2, PP5)	Yes (cosegregation analysis) and LRS. One sample could not be confirmed by second technique

ACMG, American College of Medical Genetics and Genomics; CKD, chronic kidney disease; LRS, long-read sequencing; VNTR, variable number tandem repeat.

**Table 4:** List of 28 patients reported in this study and summary of clinical information

Sample	Variant position	Alternative allele frequency (%)	Sex	Clinical diagnosis	Age at genetic diagnosis	Confirmation	Segregation in affected family members
M-1	NM_001371720.2: c.428dup; p.Ala144Serfs∗86	40%	Male	Chronic kidney disease with suspicion of ADTKD. Positive family history of nephropathy	40	Sanger	Yes
M-2	NM_001371720.2: c.428dup; p.Ala144Serfs∗86	46%	Female	Unexplained chronic kidney disease. Renal transplant at 50 y.	72	Sanger	-
M-3	NM_001371720.2: c.428dup; p.Ala144Serfs∗86	38%	Male	Unexplained chronic kidney disease. Renal transplant at 34 y.	63	Sanger	-
M-4	NM_001371720.2: c.428dup; p.Ala144Serfs∗86	41%	Female	Chronic kidney disease with suspicion of ADTKD. Positive family history of nephropathy	48	Sanger	Yes
M-5	NM_001371720.2: c.428dup; p.Ala144Serfs∗86	45%	Male	Chronic kidney disease on dialysis with suspicion of ADTKD. Positive family history of nephropathy	31	Sanger	Yes
M-6	NM_001371720.2: c.488dup; p.Ala164Serfs∗106	47%	Female	Mild renal insufficiency with family history of inherited nephropathy with renal transplantation	35	Sanger	Not performed
M-7	NM_001371720.2: c.428dup; p.Ala144Serfs∗86	40%	Male	Unexplained chronic kidney disease. Suspicion of ADTKD	35	Sanger	-
M-8	NM_001371720.2: c.428dup; p.Ala144Serfs∗86	44%	Female	Chronic kidney disease with suspicion of ADTKD. Renal transplant at 56 y. Positive family history of nephropathy	68	Sanger	Yes
M-9	NM_001371720.2: c.428dup; p.Ala144Serfs∗86	42%	Male	Unexplained chronic kidney disease	63	Sanger	-
M-10	NM_001371720.2: c.428dup; p.Ala144Serfs∗86	45%	Male	Unexplained chronic kidney disease. Suspicion of Alport syndrome. Positive family history of nephropathy	55	Sanger	Yes
M-11	NM_001371720.2: c.428dup; p.Ala144Serfs∗86	32%	Male	Chronic kidney disease with suspicion of ADTKD with onset at 44 y. Positive family history of nephropathy	74	Sanger	Yes
M-12	NM_001371720.2: c.428dup; p.Ala144Serfs∗86	40%	Male	Suspicion of Alport syndrome. Positive family history of nephropathy	53	Sanger	Yes
M-13	NM_001371720.2: c.428dup; p.Ala144Serfs∗86	34%	Male	Suspicion of ADTKD. Positive family history of nephropathy	42	Sanger	Yes
M-14	NC_000001.10:g.(155160963_155162030)insG	0.91%	Female	Chronic renal insufficiency with suspicion of ADTKD and positive family history of nephropathy	66	SNaPshot	Not performed
M-15	NC_000001.10:g.(155160963_155162030)insG	1.76%	Male	Unexplained chronic kidney disease. Renal transplant at 29 yr and positive family history of nephropathy	31	LRS	Not performed
M-16	NC_000001.10:g.(155160963_155162030)insG	1.12%	Female	Unexplained chronic kidney disease. Renal transplant at 28 yr.	44	SNaPshot	-
M-17	NC_000001.10:g.(155160963_155162030)insG	1.38%	Male	Unexplained chronic kidney disease. Suspicion of ADTKD	31	SNaPshot	-
M-18	NC_000001.10:g.(155160963_155162030)insG	0.56%	Female	Unexplained chronic kidney disease. Renal transplant at 47 yr.	53	LRS	-
M-19	NC_000001.10:g.(155160963_155162030)insG	1.05%	Male	Chronic kidney disease. Renal transplant at 27 yr. Suspicion of ADTKD	39	LRS	-
M-20	NC_000001.10:g.(155160963_155162030)insG	0.90%	Male	Unexplained chronic kidney disease. Renal transplant at 25 yr.	32	SNaPshot	-
M-21	NC_000001.10:g.(155160963_155162030)insG	0.64%	Male	Chronic kidney disease. Renal transplant at 38 yr. Suspicion of ADTKD. Positive family history of nephropathy	55	LRS	Yes
M-22	NC_000001.10:g.(155160963_155162030)insG	0.87%	Female	Unexplained chronic kidney disease with positive family history of nephropathy	51	SNaPshot	Not performed
M-23	NC_000001.10:g.(155160963_155162030)insG	1.18%	Male	Unexplained chronic kidney disease	47	SNaPshot	-
M-24	NC_000001.10:g.(155160963_155162030)insG	1.15%	Female	Chronic kidney disease with suspicion of ADTKD	58	SNaPshot	-
M-25	NC_000001.10:g.(155160963_155162030)insG	0.54%	Female	Unexplained chronic kidney disease. Dialysis at 29 yr and positive family history of nephropathy	22	SNaPshot	Not performed
M-34	NC_000001.10:g.(155160963_155162030)insT	1.41%	Male	Unexplained chronic kidney disease. Renal transplant at 54 yr.	62	Not performed	Yes
M-550	NC_000001.10:g.(155160963_155162030)insT	0.68%	Female	Chronic kidney disease diagnosed as ADTKD	32	Not performed	-
M-31	NC_000001.10:g.(155160963_155162030)insT	0.74%	Male	Chronic kidney disease with suspicion of ADTKD. Positive family history of nephropathy. Cousin of M-34	62	LRS	Yes

ADTKD, autosomal dominant tubulointerstitial kidney disease; CKD, chronic kidney disease; LRS, long-read sequencing (Pacific Biosciences); NC, NCBI genomic reference sequence; NM, NCBI mRNA reference sequence; VNTR, variable number tandem repeat.

## Discussion

Various strategies have been proposed for the analysis of variants within the VNTR*-MUC1* region, owing to the considerable challenges associated with its accurate characterization. These challenges encompass both the development of robust laboratory methodologies and the availability of analytical tools capable of reliably detecting variants with high accuracy. The heterogeneity of existing approaches, coupled with the limited expertise in this highly specialized field, has hindered the widespread adoption and implementation of technologies that enable comprehensive *MUC1* analysis in the diagnostic routine. As a consequence, the prevalence of ADTKD-*MUC1* has likely been underestimated, resulting in a substantial number of missed or delayed diagnoses.

We analyzed a large cohort of patients with CKDx using a customized SR-HTS approach targeting the *MUC1* gene, including its VNTR region, with the aim of identifying pathogenic variants. The most prevalent pathogenic variant (DupC) within the VNTR*-MUC1* region was detected in 25 samples. In addition, a rare frameshift variant (DupA) was identified in 3 patients clinically diagnosed with ADTKD, yielding an overall prevalence of ADTKD-*MUC1* in the studied sample of 5%.

The most frequent *MUC1* gene variant was a cytosine duplication within the first repeat module of the VNTR region, which can be readily detected by standard variant-calling algorithms commonly applied in next-generation sequencing analyses, including WES. In addition, the same pathogenic duplication was identified in the second repeat module in 1 sample, exhibiting sequencing characteristics comparable to those observed for insertions in the first module, with the only distinction being differences in the alternative allele frequency.

In 12 of the 25 DupC-positive patients (48%), the DupC was located within internal VNTR repeat modules that are not detectable by conventional WES or other standard next-generation sequencing approaches, because these methods typically do not provide comprehensive coverage of the full VNTR-*MUC1* region. To overcome this limitation, we enriched the region of interest using a customized library preparation strategy and implemented a dedicated bioinformatic pipeline designed to detect cytosine duplications across all VNTR repeats. This approach also enabled the screening of additional potentially pathogenic variants within the same region, including the previously reported DupA adenine duplication.^9^

Our findings suggest that additional recurrent *MUC1* variants may be underestimated, because 3 patients (10%) of frameshift-positive *MUC1* cases carried DupA variants within the VNTR region. To our knowledge, these are the first segregation data for DupA in affected family members. The DupA variant has been previously characterized and is predicted to generate a neoprotein effect similar to that described for DupC.^9^ Consistent with this model, the DupA variant is predicted to be expressed and to accumulate in the cytoplasm. Immunostaining using *MUC1* frameshift-specific antibodies represents an established approach to confirm the pathogenic consequences of frameshift variants located within the VNTR region of *MUC1*, as previously described.^20^

The variability observed in the VNTR variant ratio may be attributed to several factors. One contributing factor is sequencing read depth, which can vary between runs. However, the observed inter-run concordance supports a limited impact on the overall estimates. The predominant factor influencing ratio estimation is the inter-individual heterogeneity in VNTR repeat number, which directly affects the proportion of reads derived from each allele.

LRS enabled accurate determination of VNTR repeat length, revealing marked inter-individual variability in repeat number and, consequently, substantial differences in sequencing coverage across this region. We observed increased read depth in patients carrying a higher number of VNTR repeats, together with a lower proportion of reads attributable to the wild-type allele (unpublished data). These findings support the notion that repeat-length heterogeneity significantly influences the estimation of mutant allele proportions within the VNTR-*MUC1* region.

We explored the presence of additional frameshift variants—distinct from those reported in this study—that may have been historically overlooked. Our approach did not reveal other non-DupC/DupA variants. However, these results must be interpreted with caution to ensure the selection of the most appropriate complementary validation technique. In this context, LRS represents the most suitable method for the comprehensive detection of any mutation type within the VNTR region.

ADTKD-*MUC1* was previously called medullary cystic kidney disease type 1. However, medullary cysts are neither a common feature nor a good predictor of the disease.^21^ A more precise diagnosis includes the identification of intracellular *MUC1* deposits in distal tubules on renal biopsy using immunohistochemistry, and usually an autosomal dominant family history. We emphasize the importance of comprehensive analysis of the entire *MUC1* sequence, minimizing technical limitations, in the context of clinical ADTKD that is genetically unexplained. Innovative approaches, such as the one proposed in this study, increase the diagnostic yield for detecting *MUC1* variants. Given the highly heterogeneous and frequent nonspecific clinical presentation associated with ADTKD-*MUC1*, our findings further support the inclusion of complete *MUC1* genetic analysis in patients with CKDx, rather than restricting testing to cases with a strong clinical suspicion of ADTKD. Whereas other primary genetic causes of ADTKD—such as defects in *UMOD*, *REN*, or *HNF1B*—are frequently associated with characteristic clinical features (e.g., hyperuricemia, renal cysts, or acid-base imbalance), no specific clinical markers have been established for ADTKD-*MUC1* in the absence of a kidney biopsy.^1^ Consequently, disease progression may remain clinically silent until patients present with established CKD, especially in early adulthood.

The implementation of a customized SR-HTS strategy combined with a dedicated bioinformatic pipeline tailored to the VNTR-*MUC1* region streamlines genetic testing in patients with CKDx or suspected ADTKD, reducing diagnostic turnaround time and the overall costs associated with the sequential application of multiple techniques. Similar strategies based on SR-HTS have been proposed to simplify the detection of *MUC1* variants with cost-effective benefits.^22^ However, it is important to highlight the limitations of these approaches and how to confirm each genetic defect. Thus, Sanger sequencing was optimized for the first 3 VNTR repeat modules as a complementary method to confirm variants located in proximal repeats. Alternative approaches, such as SNaPshot or LRS, may be selectively applied when a positive signal is detected by SR-HTS, rather than being used as first-line techniques.

Although DupC remains the most prevalent variant in ADTKD-*MUC1* patients, at least 10% of affected individuals may harbor non-DupC variants, underscoring the importance of comprehensive analysis of the entire *MUC1* sequence. The lack of routine screening for non-DupC variants may therefore contribute to the proportion of unresolved ADTKD cases. However, because of the limited number of suspected positive cases, a similar validation strategy could not be applied to DupA. Therefore, the analytical validation of the SR-HTS approach can currently be established only for the DupC variant. No additional signals suggestive of other truncating variants were detected within the VNTR region.

LRS represents a reliable and comprehensive approach for the analysis of the VNTR region in the *MUC1* gene. LRS costs remain significantly higher than those for SR sequencing; therefore, this customized SR-HTS combined with optimized bioinformatic pipelines for the resolution of complex genomic regions is likely to become the most accurate and cost-effective strategy for the genetic evaluation of patients with ADTKD.

Some limitations should be acknowledged. Validation of the present SR-HTS approach is limited by the technical complexity of the VNTR region and the relatively small number of confirmatory cases; analytical validation of the SR-HTS approach was only established for DupC and the presence of alternative pathogenic alterations not captured in this study cannot be excluded. Data presented in this study allowed the establishment of a decision threshold (>0.45% allele frequency) for the confirmation of DupC variants within the VNTR region. The clear separation between the maximum level of technical noise (0.37%) and the minimum confirmed signal (0.54%) provides a robust safety margin for clinical reporting, although confirmation of the variant detected by SR-HTS by complementary techniques is always recommended. Samples falling within the 0.36%–0.54% range should be considered within an indeterminate zone requiring additional validation. The same approach was performed to evaluate the DupA variant. We were unable to obtain a reliable score for the DupA variant using the same validation strategy applied to the DupC variant, owing to the limited number of patients carrying the DupA variant (n = 3). Nevertheless, the allele frequency of DupA was markedly higher in patients than in control samples, as clearly illustrated in Figure 5. Based on these results, each variant within the VNTR of the *MUC1* gene should be detected according to different parameters as observed in our results. In addition, we did not find in our cohort any other undescribed variants in the VNTR region, although we cannot completely rule out the presence of all possible relevant variants in *MUC1*.

Our approach allowed us to diagnose more than 50% of patients with *MUC1*-ADTKD who had been negative for conventional genetic testing. Frameshift mutations in the VNTR region other than the DupC variant may be challenging to detect and confirm; therefore, a specific decision algorithm, such as the one we propose in this study, can help resolve underdiagnosed cases.

In conclusion, this study presents a suitable application of a customized SR-HTS approach for the systematic screening of *MUC1* gene variants, with particular emphasis on the detection of frameshift alterations within the VNTR region that generate pathogenic *MUC1* neoproteins associated with ADTKD-*MUC1*. Integrating this strategy into routine diagnostic workflows, together with the parallel analysis of other genes implicated in CKD and ADTKD, increases the overall diagnostic yield while reducing the technical complexity associated with the sequential application of multiple methodologies. Moreover, this approach supports a more efficient and structured decision-making process for the interpretation and validation of variants identified within the VNTR-*MUC1* region.

## Disclosure

JGA is an employee of Health in Code SL. AO’s research is supported by Comunidad de Madrid en Biomedicina P2022/BMD-7223, CIFRA_COR-CM, Instituto de Salud Carlos III (ISCIII) (PI22/00469, PI22/00050, PI21/00251, PI25/00145), ERA-PerMed-JTC2022 (SPAREKID AC22/00027), the RICORS program to RICORS2040-renal (RD24/0004/0001) co-funded by the European Union, and SPACKDc PMP21/00109, FEDER funds; COST Action PERMEDIK CA21165 supported by COST (European Cooperation in Science and Technology); PREVENTCKD Consortium (Project ID 101101220, Programme EU4H, DG/Agency HADEA); KitNewCare (Project ID 101137054, Call HORIZON-HLTH-2023-CARE-04, Programme HORIZON, DG/Agency HADEA); and PICKED (Project ID 101168626, HORIZON-MSCA-2023-DN-01-01, MSCA Doctoral Networks 2023). VPG’s research is supported by ISCIII PI22/01168 and PI25/01565, co-funded by the European Union. EAC’s research is supported by ISCIII/FEDER (PI23/00426). All other authors declared no competing interests.

## Supplementary Material

Supplementary File (PDF) **Table S1.** Supported data for DupC and DupA detection in VNTR region of MUC1 gene. The data from coverage and number of DupC- and DupA-reads belong from 537 samples. 13 samples, which harbored DupC variant in the first and second repeat were excluded from the representa on because they were detected by conventional variant callers with standard parameters of quality and alternative allele frequency. **S2.** Short-read *MUC1* analysis scripts and inputs. **S3.** Long-read *MUC1* analysis scripts and inputs.

## Footnotes

Supplementary File (PDF)
**Table S1.** Supported data for DupC and DupA detection in VNTR region of *MUC1* gene. The data from coverage and number of DupC- and DupA-reads belong from 537 samples. 13 samples, which harbored DupC variant in the first and second repeat were excluded from the representation because they were detected by conventional variant callers with standard parameters of quality and alternative allele frequency. **S2.** Short-read *MUC1* analysis scripts and inputs. **S3.** Long-read *MUC1* analysis scripts and inputs.
