# Supplementary material for: Customized MUC1 Sequencing Enables Diagnosis of Autosomal Dominant Tubulointerstitial Kidney Disease and Unexplained CKD
Source: Kidney Int Rep. 2026 Sep 25;11(12):107095. doi: 10.1016/j.ekir.2026.107095 (PMC13720252; doi:10.1016/j.ekir.2026.107095)
Supplement: Supplementary file 1 [file mmc1.pdf]

Supplementary material

Table S1: Supported data for DupC and DupA detection in VNTR region of *MUC1* gene. The data from coverage and number of DupC- and DupA-reads belong from 537 samples. 13 samples, which harbored DupC variant in the first and second repeat were excluded from the representation because they were detected by conventional variant callers with standard parameters of quality and alternative allele frequency.

Sample	Coverage VNTR	C-insertion reads	% DupC read	Confirmation
**M-14**	**34028**	**309**	**0,91**	Positive SNaPshot
**M-15**	**10597**	**187**	**1,76**	Positive LR
**M-16**	**15431**	**173**	**1,12**	Positive SNaPshot
**M-17**	**10003**	**138**	**1,38**	Positive SNaPshot
**M-18**	**20731**	**116**	**0,56**	Positive LR
**M-19**	**9249**	**97**	**1,05**	Positive LR
**M-20**	**8700**	**78**	**0,90**	Positive SNaPshot
**M-21**	**27731**	**177**	**0,64**	Positive LR
**M-22**	**18593**	**161**	**0,87**	Positive SNaPshot
**M-23**	**30230**	**358**	**1,18**	Positive SNaPshot
**M-24**	**33799**	**387**	**1,15**	Positive SNaPshot
**M-25**	**5197**	**28**	**0,54**	Positive SNaPshot
M-26	12399	41	0,33	Negative SNaPshot
M-27	24849	78	0,31	Negative SNaPshot
M-28	20046	73	0,36	Negative SNaPshot
M-29	9254	31	0,33	Negative SNaPshot
M-30	4837	7	0,14	Negative LR
M-31	8061	28	0,35	Negative SNaPshot
M-32	20969	69	0,33	Negative SNaPshot
M-33	3312	10	0,30	Negative SNaPshot
M-34	17609	37	0,21	Negative SNaPshot
M-35	16499	37	0,22	
M-36	38354	54	0,14	
M-37	46893	50	0,11	
M-38	39199	50	0,13	
M-39	38476	50	0,13	
M-40	27010	48	0,18	
M-41	24510	47	0,19	

Sample	Coverage VNTR	T-insertion reads	% DupA read	Confirmation
**M-34**	**17609**	**249**	1,41	**Family member of M-31**
**M-550**	**28806**	**197**	0,68	**Not performed**
**M-31**	**8061**	**60**	0,74	**Positive SNaPshot**
M-537	2784	3	0,11	
M-222	13208	14	0,11	
M-511	2074	2	0,10	
M-434	12040	11	0,09	
M-165	15881	14	0,09	
M-538	2540	2	0,08	
M-308	8183	7	0,09	
M-279	11534	10	0,09	
M-485	6030	5	0,08	
M-280	11235	9	0,08	
M-282	9504	8	0,08	
M-411	16127	13	0,08	
M-504	4938	4	0,08	
M-416	9675	8	0,08	
M-545	31037	23	0,07	
M-62	25354	20	0,08	
M-429	6521	5	0,08	
M-390	17427	13	0,07	
M-548	30957	21	0,07	
M-140	14842	11	0,07	
M-498	2742	2	0,07	
M-393	13733	10	0,07	
M-44	42027	30	0,07	
M-41	24510	18	0,07	
M-328	6922	5	0,07	

M-42	39764	45	0,11
M-43	23957	45	0,19
M-44	42027	44	0,10
M-45	35522	44	0,12
M-46	29710	41	0,14
M-47	28834	40	0,14
M-48	29194	39	0,13
M-49	25490	39	0,15
M-50	25230	39	0,15
M-51	12539	38	0,30
M-52	25420	37	0,15
M-53	23860	36	0,15
M-54	29517	35	0,12
M-55	24329	35	0,14
M-56	32819	34	0,10
M-57	30007	34	0,11
M-58	26995	34	0,13
M-59	22899	34	0,15
M-60	22688	34	0,15
M-61	22025	34	0,15
M-62	25354	33	0,13
M-63	23139	33	0,14
M-64	17134	33	0,19
M-65	31366	32	0,10
M-66	24939	32	0,13
M-67	24568	32	0,13
M-68	22925	32	0,14
M-69	20132	32	0,16
M-70	19379	32	0,17
M-71	17071	32	0,19
M-72	30977	31	0,10
M-73	25436	31	0,12
M-74	21428	31	0,14
M-75	17182	31	0,18
M-76	21492	30	0,14
M-77	21088	30	0,14
M-78	20943	30	0,14

M-343	8347	6	0,07
M-267	12643	9	0,07
M-464	8121	6	0,07
M-423	7331	5	0,07
M-219	15475	11	0,07
M-283	8755	6	0,07
M-486	5825	4	0,07
M-105	14900	10	0,07
M-108	8972	6	0,07
M-36	38354	26	0,07
M-543	27930	17	0,06
M-58	26995	18	0,07
M-27	24849	15	0,06
M-258	16849	11	0,07
M-421	7554	5	0,07
M-22	18593	11	0,06
M-19	9249	6	0,06
M-191	12465	8	0,06
M-294	12588	8	0,06
M-547	17192	10	0,06
M-401	9479	6	0,06
M-265	14281	9	0,06
M-220	14126	9	0,06
M-368	6299	4	0,06
M-348	16028	10	0,06
M-519	3233	2	0,06
M-518	3259	2	0,06
M-413	11162	7	0,06
M-358	9411	6	0,06
M-139	16159	10	0,06
M-408	4941	3	0,06
M-101	17880	11	0,06
M-154	13143	8	0,06
M-24	33799	18	0,05
M-127	18433	11	0,06
M-372	15236	9	0,06
M-21	27731	18	0,06

M-79	18617	30	0,16
M-80	17568	30	0,17
M-81	28459	29	0,10
M-82	22425	29	0,13
M-83	22375	29	0,13
M-84	22201	29	0,13
M-85	13916	29	0,21
M-86	36446	28	0,08
M-87	25321	28	0,11
M-88	23679	28	0,12
M-89	23567	28	0,12
M-90	20891	28	0,13
M-91	19596	28	0,14
M-92	17279	28	0,16
M-93	16033	28	0,17
M-94	15512	28	0,18
M-95	15148	28	0,18
M-96	13456	28	0,21
M-97	28597	27	0,09
M-98	21842	27	0,12
M-99	20134	27	0,13
M-100	19152	27	0,14
M-101	17880	27	0,15
M-102	16644	27	0,16
M-103	15866	27	0,17
M-104	15852	27	0,17
M-105	14900	27	0,18
M-106	14031	27	0,19
M-107	12158	27	0,22
M-108	8972	27	0,30
M-109	35709	26	0,07
M-110	34475	26	0,08
M-111	32213	26	0,08
M-112	24862	26	0,10
M-113	23860	26	0,11
M-114	18094	26	0,14
M-115	15622	26	0,17

M-468	6808	4	0,06
M-221	13731	8	0,06
M-166	15330	9	0,06
M-496	3474	2	0,06
M-175	13838	8	0,06
M-146	17344	10	0,06
M-503	5306	3	0,06
M-276	14216	8	0,06
M-195	19635	11	0,06
M-403	8792	5	0,06
M-517	3629	2	0,06
M-338	10477	6	0,06
M-287	19977	11	0,06
M-512	1912	1	0,05
M-206	10805	6	0,06
M-111	32213	18	0,06
M-444	5371	3	0,06
M-336	12572	7	0,06
M-144	20056	11	0,05
M-295	12576	7	0,06
M-425	7192	4	0,06
M-48	29194	16	0,05
M-266	12796	7	0,05
M-215	18116	10	0,06
M-28	20046	10	0,05
M-172	18658	10	0,05
M-312	16813	9	0,05
M-185	14934	8	0,05
M-263	15158	8	0,05
M-223	13139	7	0,05
M-319	11304	6	0,05
M-323	9503	5	0,05
M-357	9536	5	0,05
M-46	29710	16	0,05
M-79	18617	10	0,05
M-174	15128	8	0,05
M-60	22688	12	0,05

M-116	14935	26	0,17
M-117	14732	26	0,18
M-118	35526	25	0,07
M-119	31875	25	0,08
M-120	24941	25	0,10
M-121	23340	25	0,11
M-122	20228	25	0,12
M-123	20078	25	0,12
M-124	20070	25	0,12
M-125	19464	25	0,13
M-126	18543	25	0,13
M-127	18433	25	0,14
M-128	14443	25	0,17
M-129	24497	24	0,10
M-130	23440	24	0,10
M-131	23268	24	0,10
M-132	23202	24	0,10
M-133	22155	24	0,11
M-134	22088	24	0,11
M-135	21621	24	0,11
M-136	20533	24	0,12
M-137	18504	24	0,13
M-138	18263	24	0,13
M-139	16159	24	0,15
M-140	14842	24	0,16
M-141	14034	24	0,17
M-142	21863	23	0,11
M-143	20833	23	0,11
M-144	20056	23	0,11
M-145	18788	23	0,12
M-146	17344	23	0,13
M-147	15551	23	0,15
M-148	15163	23	0,15
M-149	15163	23	0,15
M-150	13988	23	0,16
M-151	13810	23	0,17
M-152	13801	23	0,17

M-188	13572	7	0,05
M-156	25233	13	0,05
M-94	15512	8	0,05
M-76	21492	11	0,05
M-262	15484	8	0,05
M-230	9874	5	0,05
M-491	3903	2	0,05
M-386	7825	4	0,05
M-255	7911	4	0,05
M-326	7746	4	0,05
M-286	22259	11	0,05
M-231	9791	5	0,05
M-321	10257	5	0,05
M-176	12208	6	0,05
M-97	28597	14	0,05
M-345	6177	3	0,05
M-30	4837	3	0,06
M-384	8176	4	0,05
M-349	14523	7	0,05
M-186	14811	7	0,05
M-50	25230	12	0,05
M-143	20833	10	0,05
M-23	30230	13	0,04
M-32	20969	9	0,04
M-225	12706	6	0,05
M-91	19596	9	0,05
M-232	8413	4	0,05
M-189	13076	6	0,05
M-80	17568	8	0,05
M-72	30977	14	0,05
M-236	22551	10	0,04
M-38	39199	18	0,05
M-383	8740	4	0,05
M-313	13492	6	0,04
M-310	6676	3	0,04
M-332	13477	6	0,04
M-362	8789	4	0,05

M-153	13232	23	0,17
M-154	13143	23	0,17
M-155	12343	23	0,19
M-156	25233	22	0,09
M-157	22097	22	0,10
M-158	20904	22	0,11
M-159	20590	22	0,11
M-160	18184	22	0,12
M-161	17341	22	0,13
M-162	17088	22	0,13
M-163	16508	22	0,13
M-164	16034	22	0,14
M-165	15881	22	0,14
M-166	15330	22	0,14
M-167	14499	22	0,15
M-168	13070	22	0,17
M-169	32643	21	0,06
M-170	20481	21	0,10
M-171	19276	21	0,11
M-172	18658	21	0,11
M-173	17109	21	0,12
M-174	15128	21	0,14
M-175	13838	21	0,15
M-176	12208	21	0,17
M-177	12041	21	0,17
M-178	11852	21	0,18
M-179	11779	21	0,18
M-180	11601	21	0,18
M-181	25150	20	0,08
M-182	18690	20	0,11
M-183	15125	20	0,13
M-184	15105	20	0,13
M-185	14934	20	0,13
M-186	14811	20	0,14
M-187	13788	20	0,15
M-188	13572	20	0,15
M-189	13076	20	0,15

M-360	8880	4	0,05
M-549	10325	5	0,05
M-329	6609	3	0,05
M-99	20134	9	0,04
M-160	18184	8	0,04
M-476	4611	2	0,04
M-217	16139	7	0,04
M-274	15963	7	0,04
M-59	22899	10	0,04
M-247	13600	6	0,04
M-131	23268	10	0,04
M-392	14011	6	0,04
M-56	32819	14	0,04
M-510	2371	1	0,04
M-377	11706	5	0,04
M-316	11689	5	0,04
M-35	16499	7	0,04
M-130	23440	10	0,04
M-205	11842	5	0,04
M-106	14031	6	0,04
M-179	11779	5	0,04
M-539	2444	1	0,04
M-128	14443	6	0,04
M-303	9633	4	0,04
M-177	12041	5	0,04
M-417	9477	4	0,04
M-202	14184	6	0,04
M-424	7275	3	0,04
M-264	14755	6	0,04
M-288	19765	8	0,04
M-243	15011	6	0,04
M-340	9877	4	0,04
M-112	24862	10	0,04
M-116	14935	6	0,04
M-52	25420	10	0,04
M-71	17071	7	0,04
M-184	15105	6	0,04

M-190	13025	20	0,15
M-191	12465	20	0,16
M-192	12321	20	0,16
M-193	19318	10	0,02
M-194	21680	19	0,09
M-195	19635	19	0,10
M-196	18966	19	0,10
M-197	18964	19	0,10
M-198	18449	19	0,10
M-199	18142	19	0,10
M-200	16290	19	0,12
M-201	15801	19	0,12
M-202	14184	19	0,13
M-203	13939	19	0,14
M-204	12441	19	0,15
M-205	11842	19	0,16
M-206	10805	19	0,18
M-207	10182	19	0,19
M-208	10080	19	0,19
M-209	28540	18	0,06
M-210	25767	18	0,07
M-211	23374	18	0,08
M-212	21263	18	0,08
M-213	21068	18	0,09
M-214	18840	18	0,10
M-215	18116	18	0,10
M-216	16938	18	0,11
M-217	16139	18	0,11
M-218	15499	18	0,12
M-219	15475	18	0,12
M-220	14126	18	0,13
M-221	13731	18	0,13
M-222	13208	18	0,14
M-223	13139	18	0,14
M-224	12948	18	0,14
M-225	12706	18	0,14
M-226	12375	18	0,15

M-422	7371	3	0,04
M-122	20228	8	0,04
M-25	5197	2	0,04
M-207	10182	4	0,04
M-63	23139	9	0,04
M-68	22925	9	0,04
M-193	19318	7	0,04
M-389	5152	2	0,04
M-209	28540	11	0,04
M-147	15551	6	0,04
M-16	15431	6	0,04
M-410	20650	8	0,04
M-521	2664	1	0,04
M-115	15622	6	0,04
M-291	15736	6	0,04
M-126	18543	7	0,04
M-325	8074	3	0,04
M-190	13025	5	0,04
M-104	15852	6	0,04
M-452	10708	4	0,04
M-250	10606	4	0,04
M-103	15866	6	0,04
M-420	8077	3	0,04
M-542	17598	7	0,04
M-246	13777	5	0,04
M-371	16827	6	0,04
M-61	22025	8	0,04
M-55	24329	9	0,04
M-277	13845	5	0,04
M-284	8243	3	0,04
M-443	5603	2	0,04
M-471	5939	2	0,03
M-152	13801	5	0,04
M-134	22088	8	0,04
M-125	19464	7	0,04
M-163	16508	6	0,04
M-83	22375	8	0,04

M-227	12318	18	0,15
M-228	12295	18	0,15
M-229	10760	18	0,17
M-230	9874	18	0,18
M-231	9791	18	0,18
M-232	8413	18	0,21
M-233	37365	17	0,05
M-234	25699	17	0,07
M-235	25597	17	0,07
M-236	22551	17	0,08
M-237	19600	17	0,09
M-238	18609	17	0,09
M-239	18124	17	0,09
M-240	17518	17	0,10
M-241	17504	17	0,10
M-242	16203	17	0,10
M-243	15011	17	0,11
M-244	14594	17	0,12
M-245	13912	17	0,12
M-246	13777	17	0,12
M-247	13600	17	0,13
M-248	12323	17	0,14
M-249	11887	17	0,14
M-250	10606	17	0,16
M-251	10128	17	0,17
M-252	10017	17	0,17
M-253	9887	17	0,17
M-254	8931	17	0,19
M-255	7911	17	0,21
M-256	20465	16	0,08
M-257	17573	16	0,09
M-258	16849	16	0,09
M-259	16042	16	0,10
M-260	15628	16	0,10
M-261	15511	16	0,10
M-262	15484	16	0,10
M-263	15158	16	0,11

M-278	13789	5	0,04
M-102	16644	6	0,04
M-120	24941	9	0,04
M-81	28459	10	0,04
M-307	8350	3	0,04
M-409	23183	8	0,03
M-203	13939	5	0,04
M-29	9254	3	0,03
M-404	8359	3	0,04
M-311	29111	10	0,03
M-515	5773	2	0,03
M-546	18685	6	0,03
M-124	20070	7	0,03
M-210	25767	9	0,03
M-317	11642	4	0,03
M-69	20132	7	0,03
M-211	23374	8	0,03
M-388	5812	2	0,03
M-136	20533	7	0,03
M-520	2989	1	0,03
M-109	35709	12	0,03
M-249	11887	4	0,03
M-78	20943	7	0,03
M-442	5854	2	0,03
M-57	30007	10	0,03
M-268	12106	4	0,03
M-114	18094	6	0,03
M-117	14732	5	0,03
M-507	3230	1	0,03
M-396	12099	4	0,03
M-77	21088	7	0,03
M-435	12020	4	0,03
M-226	12375	4	0,03
M-155	12343	4	0,03
M-342	9249	3	0,03
M-296	12369	4	0,03
M-508	3041	1	0,03

M-264	14755	16	0,11
M-265	14281	16	0,11
M-266	12796	16	0,13
M-267	12643	16	0,13
M-268	12106	16	0,13
M-269	11874	16	0,13
M-270	11621	16	0,14
M-271	10149	16	0,16
M-272	6211	16	0,26
M-273	18794	15	0,08
M-274	15963	15	0,09
M-275	14668	15	0,10
M-276	14216	15	0,11
M-277	13845	15	0,11
M-278	13789	15	0,11
M-279	11534	15	0,13
M-280	11235	15	0,13
M-281	11001	15	0,14
M-282	9504	15	0,16
M-283	8755	15	0,17
M-284	8243	15	0,18
M-285	5991	15	0,25
M-286	22259	14	0,06
M-287	19977	14	0,07
M-288	19765	14	0,07
M-289	18967	14	0,07
M-290	15780	14	0,09
M-291	15736	14	0,09
M-292	14899	14	0,09
M-293	13483	14	0,10
M-294	12588	14	0,11
M-295	12576	14	0,11
M-296	12369	14	0,11
M-297	12170	14	0,12
M-298	11874	14	0,12
M-299	11518	14	0,12
M-300	10562	14	0,13

M-67	24568	8	0,03
M-374	12495	4	0,03
M-198	18449	6	0,03
M-182	18690	6	0,03
M-273	18794	6	0,03
M-402	9328	3	0,03
M-87	25321	8	0,03
M-527	3391	1	0,03
M-118	35526	11	0,03
M-302	9766	3	0,03
M-197	18964	6	0,03
M-93	16033	5	0,03
M-322	9756	3	0,03
M-242	16203	5	0,03
M-380	9881	3	0,03
M-234	25699	8	0,03
M-399	9693	3	0,03
M-367	6478	2	0,03
M-70	19379	6	0,03
M-132	23202	7	0,03
M-54	29517	9	0,03
M-153	13232	4	0,03
M-366	6541	2	0,03
M-123	20078	6	0,03
M-456	6694	2	0,03
M-354	10198	3	0,03
M-379	10067	3	0,03
M-437	10032	3	0,03
M-113	23860	7	0,03
M-96	13456	4	0,03
M-339	9965	3	0,03
M-251	10128	3	0,03
M-238	18609	6	0,03
M-394	13447	4	0,03
M-293	13483	4	0,03
M-480	3486	1	0,03
M-479	3512	1	0,03

M-301	9783	14	0,14
M-302	9766	14	0,14
M-303	9633	14	0,15
M-304	9155	14	0,15
M-305	9044	14	0,15
M-306	8465	14	0,17
M-307	8350	14	0,17
M-308	8183	14	0,17
M-309	7759	14	0,18
M-310	6676	14	0,21
M-311	29111	13	0,04
M-312	16813	13	0,08
M-313	13492	13	0,10
M-314	12259	13	0,11
M-315	11690	13	0,11
M-316	11689	13	0,11
M-317	11642	13	0,11
M-318	11532	13	0,11
M-319	11304	13	0,12
M-320	11172	13	0,12
M-321	10257	13	0,13
M-322	9756	13	0,13
M-323	9503	13	0,14
M-324	8911	13	0,15
M-325	8074	13	0,16
M-326	7746	13	0,17
M-327	7292	13	0,18
M-328	6922	13	0,19
M-329	6609	13	0,20
M-330	14365	12	0,08
M-331	13507	12	0,09
M-332	13477	12	0,09
M-333	13441	12	0,09
M-334	13209	12	0,09
M-335	12573	12	0,10
M-336	12572	12	0,10
M-337	11844	12	0,10

M-245	13912	4	0,03
M-187	13788	4	0,03
M-346	17356	5	0,03
M-119	31875	9	0,03
M-240	17518	5	0,03
M-151	13810	4	0,03
M-426	7009	2	0,03
M-455	7076	2	0,03
M-494	3526	1	0,03
M-135	21621	6	0,03
M-497	3170	1	0,03
M-467	7068	2	0,03
M-169	32643	9	0,03
M-181	25150	7	0,03
M-516	3714	1	0,03
M-327	7292	2	0,03
M-244	14594	4	0,03
M-373	14635	4	0,03
M-281	11001	3	0,03
M-477	3937	1	0,03
M-347	17203	5	0,03
M-133	22155	6	0,03
M-451	10911	3	0,03
M-292	14899	4	0,03
M-492	3775	1	0,03
M-26	12399	3	0,02
M-14	34028	9	0,03
M-478	3867	1	0,03
M-121	23340	6	0,03
M-40	27010	7	0,03
M-352	11764	3	0,03
M-269	11874	3	0,03
M-45	35522	9	0,03
M-337	11844	3	0,03
M-178	11852	3	0,03
M-315	11690	3	0,03
M-86	36446	9	0,02

M-338	10477	12	0,11
M-339	9965	12	0,12
M-340	9877	12	0,12
M-341	9466	12	0,13
M-342	9249	12	0,13
M-343	8347	12	0,14
M-344	7411	12	0,16
M-345	6177	12	0,19
M-346	17356	11	0,06
M-347	17203	11	0,06
M-348	16028	11	0,07
M-349	14523	11	0,08
M-350	14007	11	0,08
M-351	12537	11	0,09
M-352	11764	11	0,09
M-353	11003	11	0,10
M-354	10198	11	0,11
M-355	10063	11	0,11
M-356	9575	11	0,11
M-357	9536	11	0,12
M-358	9411	11	0,12
M-359	9395	11	0,12
M-360	8880	11	0,12
M-361	8854	11	0,12
M-362	8789	11	0,13
M-363	8677	11	0,13
M-364	8536	11	0,13
M-365	7485	11	0,15
M-366	6541	11	0,17
M-367	6478	11	0,17
M-368	6299	11	0,17
M-369	6192	11	0,18
M-370	5892	11	0,19
M-371	16827	10	0,06
M-372	15236	10	0,07
M-373	14635	10	0,07
M-374	12495	10	0,08

M-376	12082	3	0,02
M-525	4060	1	0,02
M-164	16034	4	0,02
M-42	39764	10	0,03
M-107	12158	3	0,02
M-448	3987	1	0,03
M-129	24497	6	0,02
M-297	12170	3	0,02
M-228	12295	3	0,02
M-385	8129	2	0,02
M-66	24939	6	0,02
M-364	8536	2	0,02
M-212	21263	5	0,02
M-490	4281	1	0,02
M-37	46893	11	0,02
M-213	21068	5	0,02
M-110	34475	8	0,02
M-173	17109	4	0,02
M-162	17088	4	0,02
M-514	8679	2	0,02
M-524	4376	1	0,02
M-216	16938	4	0,02
M-161	17341	4	0,02
M-20	8700	2	0,02
M-241	17504	4	0,02
M-489	4509	1	0,02
M-333	13441	3	0,02
M-157	22097	5	0,02
M-361	8854	2	0,02
M-475	4811	1	0,02
M-239	18124	4	0,02
M-324	8911	2	0,02
M-450	12902	3	0,02
M-544	14867	3	0,02
M-137	18504	4	0,02
M-199	18142	4	0,02
M-138	18263	4	0,02

M-375	12420	10	0,08
M-376	12082	10	0,08
M-377	11706	10	0,09
M-378	10500	10	0,10
M-379	10067	10	0,10
M-380	9881	10	0,10
M-381	9739	10	0,10
M-382	8986	10	0,11
M-383	8740	10	0,11
M-384	8176	10	0,12
M-385	8129	10	0,12
M-386	7825	10	0,13
M-387	7355	10	0,14
M-388	5812	10	0,17
M-389	5152	10	0,19
M-390	17427	9	0,05
M-391	14085	9	0,06
M-392	14011	9	0,06
M-393	13733	9	0,07
M-394	13447	9	0,07
M-395	12810	9	0,07
M-396	12099	9	0,07
M-397	11115	9	0,08
M-398	10873	9	0,08
M-399	9693	9	0,09
M-400	9542	9	0,09
M-401	9479	9	0,09
M-402	9328	9	0,10
M-403	8792	9	0,10
M-404	8359	9	0,11
M-405	7703	9	0,12
M-406	6624	9	0,14
M-407	5064	9	0,18
M-408	4941	9	0,18
M-409	23183	8	0,03
M-410	20650	8	0,04
M-411	16127	8	0,05
M-382	8986	2	0,02
M-304	9155	2	0,02
M-419	9108	2	0,02
M-418	9380	2	0,02
M-196	18966	4	0,02
M-89	23567	5	0,02
M-289	18967	4	0,02
M-233	37365	8	0,02
M-145	18788	4	0,02
M-88	23679	5	0,02
M-171	19276	4	0,02
M-356	9575	2	0,02
M-453	9614	2	0,02
M-39	38476	8	0,02
M-53	23860	5	0,02
M-47	28834	6	0,02
M-167	14499	3	0,02
M-271	10149	2	0,02
M-183	15125	3	0,02
M-208	10080	2	0,02
M-460	5072	1	0,02
M-474	4973	1	0,02
M-256	20465	4	0,02
M-218	15499	3	0,02
M-73	25436	5	0,02
M-148	15163	3	0,02
M-148	15163	3	0,02
M-95	15148	3	0,02
M-159	20590	4	0,02
M-170	20481	4	0,02
M-487	5164	1	0,02
M-355	10063	2	0,02
M-65	31366	6	0,02
M-300	10562	2	0,02
M-201	15801	3	0,02
M-462	10805	2	0,02
M-259	16042	3	0,02

M-412	12630	8	0,06
M-413	11162	8	0,07
M-414	10895	8	0,07
M-415	10451	8	0,08
M-416	9675	8	0,08
M-417	9477	8	0,08
M-418	9380	8	0,09
M-419	9108	8	0,09
M-420	8077	8	0,10
M-421	7554	8	0,11
M-422	7371	8	0,11
M-423	7331	8	0,11
M-424	7275	8	0,11
M-425	7192	8	0,11
M-426	7009	8	0,11
M-427	6976	8	0,11
M-428	6678	8	0,12
M-429	6521	8	0,12
M-430	6251	8	0,13
M-431	5952	8	0,13
M-432	4858	8	0,16
M-433	12439	7	0,06
M-434	12040	7	0,06
M-435	12020	7	0,06
M-436	10411	7	0,07
M-437	10032	7	0,07
M-438	9242	7	0,08
M-439	8971	7	0,08
M-440	6999	7	0,10
M-441	6635	7	0,11
M-442	5854	7	0,12
M-443	5603	7	0,12
M-444	5371	7	0,13
M-445	4786	7	0,15
M-446	4511	7	0,16
M-447	4054	7	0,17
M-448	3987	7	0,18

M-98	21842	4	0,02
M-142	21863	4	0,02
M-74	21428	4	0,02
M-194	21680	4	0,02
M-229	10760	2	0,02
M-473	5404	1	0,02
M-414	10895	2	0,02
M-397	11115	2	0,02
M-472	5543	1	0,02
M-82	22425	4	0,02
M-270	11621	2	0,02
M-75	17182	3	0,02
M-318	11532	2	0,02
M-180	11601	2	0,02
M-257	17573	3	0,02
M-92	17279	3	0,02
M-43	23957	4	0,02
M-470	6071	1	0,02
M-433	12439	2	0,02
M-272	6211	1	0,02
M-248	12323	2	0,02
M-369	6192	1	0,02
M-375	12420	2	0,02
M-457	6352	1	0,02
M-227	12318	2	0,02
M-351	12537	2	0,02
M-51	12539	2	0,02
M-430	6251	1	0,02
M-412	12630	2	0,02
M-235	25597	4	0,02
M-49	25490	4	0,02
M-469	6386	1	0,02
M-168	13070	2	0,02
M-334	13209	2	0,02
M-441	6635	1	0,02
M-428	6678	1	0,01
M-406	6624	1	0,02

M-449	3748	7	0,19
M-450	12902	6	0,05
M-451	10911	6	0,05
M-452	10708	6	0,06
M-453	9614	6	0,06
M-454	9229	6	0,07
M-455	7076	6	0,08
M-456	6694	6	0,09
M-457	6352	6	0,09
M-458	5537	6	0,11
M-459	5083	6	0,12
M-460	5072	6	0,12
M-461	4773	6	0,13
M-462	10805	5	0,05
M-463	10076	5	0,05
M-464	8121	5	0,06
M-465	7880	5	0,06
M-466	7442	5	0,07
M-467	7068	5	0,07
M-468	6808	5	0,07
M-469	6386	5	0,08
M-470	6071	5	0,08
M-471	5939	5	0,08
M-472	5543	5	0,09
M-473	5404	5	0,09
M-474	4973	5	0,10
M-475	4811	5	0,10
M-476	4611	5	0,11
M-477	3937	5	0,13
M-478	3867	5	0,13
M-479	3512	5	0,14
M-480	3486	5	0,14
M-481	2730	5	0,18
M-482	2144	5	0,23
M-483	10618	4	0,04
M-484	6075	4	0,07
M-485	6030	4	0,07

M-331	13507	2	0,01
M-427	6976	1	0,01
M-158	20904	3	0,01
M-85	13916	2	0,01
M-150	13988	2	0,01
M-440	6999	1	0,01
M-391	14085	2	0,01
M-350	14007	2	0,01
M-275	14668	2	0,01
M-84	22201	3	0,01
M-309	7759	1	0,01
M-405	7703	1	0,01
M-465	7880	1	0,01
M-290	15780	2	0,01
M-200	16290	2	0,01
M-64	17134	2	0,01
M-454	9229	1	0,01
M-439	8971	1	0,01
M-254	8931	1	0,01
M-438	9242	1	0,01
M-359	9395	1	0,01
M-463	10076	1	0,01
M-214	18840	2	0,01
M-100	19152	2	0,01
M-341	9466	1	0,01
M-378	10500	1	0,01
M-90	20891	2	0,01
M-18	20731	2	0,01
M-436	10411	1	0,01
M-17	10003	1	0,01
M-483	10618	1	0,01
M-15	10597	1	0,01
M-320	11172	1	0,01
M-299	11518	1	0,01
M-314	12259	1	0,01
M-335	12573	1	0,01
M-224	12948	1	0,01

M-486	5825	4	0,07
M-487	5164	4	0,08
M-488	4543	4	0,09
M-489	4509	4	0,09
M-490	4281	4	0,09
M-491	3903	4	0,10
M-492	3775	4	0,11
M-493	3719	4	0,11
M-494	3526	4	0,11
M-495	3524	4	0,11
M-496	3474	4	0,12
M-497	3170	4	0,13
M-498	2742	4	0,15
M-499	2693	4	0,15
M-500	2145	4	0,19
M-501	1968	4	0,20
M-502	7998	3	0,04
M-503	5306	3	0,06
M-504	4938	3	0,06
M-505	4818	3	0,06
M-506	4583	3	0,07
M-507	3230	3	0,09
M-508	3041	3	0,10
M-509	2797	3	0,11
M-510	2371	3	0,13
M-511	2074	3	0,14
M-512	1912	3	0,16
M-513	1859	3	0,16
M-514	8679	2	0,02
M-515	5773	2	0,03
M-516	3714	2	0,05
M-517	3629	2	0,06
M-518	3259	2	0,06
M-519	3233	2	0,06
M-520	2989	2	0,07
M-521	2664	2	0,08
M-522	2653	2	0,08

M-141	14034	1	0,01
M-330	14365	1	0,01
M-261	15511	1	0,01
M-260	15628	1	0,01
M-237	19600	1	0,01
M-285	5991	0	0,00
M-482	2144	0	0,00
M-501	1968	0	0,00
M-449	3748	0	0,00
M-370	5892	0	0,00
M-500	2145	0	0,00
M-481	2730	0	0,00
M-407	5064	0	0,00
M-447	4054	0	0,00
M-253	9887	0	0,00
M-252	10017	0	0,00
M-306	8465	0	0,00
M-432	4858	0	0,00
M-344	7411	0	0,00
M-513	1859	0	0,00
M-446	4511	0	0,00
M-305	9044	0	0,00
M-204	12441	0	0,00
M-499	2693	0	0,00
M-365	7485	0	0,00
M-445	4786	0	0,00
M-301	9783	0	0,00
M-387	7355	0	0,00
M-431	5952	0	0,00
M-363	8677	0	0,00
M-461	4773	0	0,00
M-459	5083	0	0,00
M-298	11874	0	0,00
M-495	3524	0	0,00
M-458	5537	0	0,00
M-493	3719	0	0,00
M-509	2797	0	0,00

M-523	2491	2	0,08
M-524	4376	1	0,02
M-525	4060	1	0,02
M-526	3745	1	0,03
M-527	3391	1	0,03
M-528	3218	1	0,03
M-529	3178	1	0,03
M-530	3121	1	0,03
M-531	2808	1	0,04
M-532	2609	1	0,04
M-533	2515	1	0,04
M-534	1911	1	0,05
M-535	1839	1	0,05
M-536	3477	0	0,00
M-537	2784	0	0,00
M-538	2540	0	0,00
M-539	2444	0	0,00
M-540	1267	0	0,00
M-541	292	0	0,00
M-542	17598	29	0,16
M-543	27930	30	0,11
M-544	14867	23	0,15
M-545	31037	23	0,07
M-546	18685	22	0,12
M-547	17192	27	0,16
M-548	30957	33	0,11
M-549	10325	20	0,19
M-550	28806	13	0,05

M-381	9739	0	0,00
M-353	11003	0	0,00
M-400	9542	0	0,00
M-488	4543	0	0,00
M-398	10873	0	0,00
M-523	2491	0	0,00
M-415	10451	0	0,00
M-522	2653	0	0,00
M-395	12810	0	0,00
M-466	7442	0	0,00
M-484	6075	0	0,00
M-506	4583	0	0,00
M-505	4818	0	0,00
M-535	1839	0	0,00
M-534	1911	0	0,00
M-533	2515	0	0,00
M-532	2609	0	0,00
M-502	7998	0	0,00
M-531	2808	0	0,00
M-530	3121	0	0,00
M-529	3178	0	0,00
M-528	3218	0	0,00
M-526	3745	0	0,00
M-536	3477	0	0,00
M-541	292	0	0,00
M-540	1267	0	0,00
M-192	12321	3	0,02
M-33	3312	0	0,00

S2: Short-read MUC1 analysis scripts and inputs

This appendix contains the short-read analysis script and the BAM list used as input for the batch workflow.

counter_short_reads.py

File path: short_reads/counter_short_reads.py

```
import argparse
import glob
import os
from collections import Counter
from difflib import SequenceMatcher

import pysam

# ----------------------------------------------------
# CONFIG
# ----------------------------------------------------

REGION_CHR = "chr1"
REGION_START = 155160978
REGION_END = 155162170
CANDIDATE_LEN = [60, 59, 61, 58, 62, 57, 63]

# Motivo obligatorio
ANCHOR = "TGGGGGGGCGGT"
ANCHOR2 = "GGAGCCCGGGGC"

# Referencia WT (~60 bp)
WT_REF = (
    "TGGGGGGGCGGT"
    "GGAGCCCGGGGCCGGCCTGGTGTCCGGGGCCGAGGTGACACCGTGGGC"
)
WT_REF2 = (
    "GGAGCCCGGGGC"
    "CGGCCTGGTGTCCGGGGCCGAGGTGACACCGTGGGCTGGGGGGGCGGT"
)

WT_LEN = len(WT_REF)
WT_LEN2 = len(WT_REF2)

# Umbral mínimo de similitud
SIM_THRESHOLD = 0.90

# ----------------------------------------------------
# FUNCIONES
# ----------------------------------------------------


def similarity(a, b):
    return SequenceMatcher(None, a, b).ratio()


def classify_length(length, reference_length):
    if length == reference_length:
        return "WT"

    if length < reference_length:
        return f"DEL_{reference_length-length}"

    return f"INS_{length-reference_length}"
```

```
def find_anchor_positions(seq, anchor):
    positions = []
    start = 0
    while True:
        pos = seq.find(anchor, start)
        if pos == -1:
            break
        positions.append(pos)
        start = pos + 1
    return positions


def select_best_fragment(seq, anchor_positions, reference, candidate_lens, sim_threshold,
reference_name):
    best_result = None

    for idx in range(len(anchor_positions) - 1):
        anchor_pos = anchor_positions[idx]
        next_anchor = anchor_positions[idx + 1]

        frag = seq[anchor_pos:next_anchor]
        length = len(frag)

        if length not in candidate_lens:
            continue

        sim = similarity(frag, reference)

        if sim < sim_threshold:
            continue

        result = {
            "length": length,
            "sim": sim,
            "frag": frag,
            "category": classify_length(length, len(reference)),
            "reference": reference_name,
        }

        if best_result is None or sim > best_result["sim"]:
            best_result = result

    return best_result


def compare_reference_results(result_a, result_b):
    if result_a is None:
        return result_b
    if result_b is None:
        return result_a

    dist_a = abs(result_a["length"] - 60)
    dist_b = abs(result_b["length"] - 60)

    if dist_a > dist_b:
        return result_a
    if dist_b > dist_a:
        return result_b

    if result_a["sim"] >= result_b["sim"]:
        return result_a
    return result_b


def process_read(seq, read_name, reads_out_handle, class_counter, length_counter):
    anchor_positions = find_anchor_positions(seq, ANCHOR)
    anchor2_positions = find_anchor_positions(seq, ANCHOR2)

    if not anchor_positions and not anchor2_positions:
        return 0, 0, None
```

```
    if reads_out_handle is not None:

reads_out_handle.write(f"{read_name}\tanchors={anchor_positions}\tanchor2={anchor2_positions}\
t{seq}\n")

    result = None

    if anchor_positions and not anchor2_positions:
        result = select_best_fragment(seq, anchor_positions, WT_REF, CANDIDATE_LEN,
SIM_THRESHOLD, reference_name="WT_REF")
    elif anchor2_positions and not anchor_positions:
        result = select_best_fragment(seq, anchor2_positions, WT_REF2, CANDIDATE_LEN,
SIM_THRESHOLD, reference_name="WT_REF2")
    elif anchor_positions and anchor2_positions:
        result_a = select_best_fragment(seq, anchor_positions, WT_REF, CANDIDATE_LEN,
SIM_THRESHOLD, reference_name="WT_REF")
        result_b = select_best_fragment(seq, anchor2_positions, WT_REF2, CANDIDATE_LEN,
SIM_THRESHOLD, reference_name="WT_REF2")
        result = compare_reference_results(result_a, result_b)

    if not result or result["sim"] < SIM_THRESHOLD:
        return 0, 0, None

    length_counter[result["length"]] += 1
    class_counter[result["category"]] += 1
    return 1, result["length"], result


def extract_sample_name(bam_path):
    if not bam_path:
        return "sample"

    bam_name = os.path.basename(bam_path)
    stem = os.path.splitext(bam_name)[0]
    parent_dir = os.path.basename(os.path.dirname(bam_path))

    candidate = parent_dir or stem
    candidate = candidate.replace("_DRAGEN", "")

    if candidate.startswith("S") and "L" in candidate:
        candidate = candidate.split("L", 1)[0]
    elif "_" in stem:
        candidate = stem.split("_", 1)[0]

    if not candidate:
        candidate = stem

    return candidate


def str2bool(value):
    if isinstance(value, bool):
        return value
    if value is None:
        return True
    if str(value).lower() in {"1", "true", "yes", "y", "t"}:
        return True
    if str(value).lower() in {"0", "false", "no", "n", "f"}:
        return False
    raise argparse.ArgumentTypeError(f"Boolean value expected, got: {value}")


def parse_args():
    parser = argparse.ArgumentParser(description="Analyze short-read MUC1 repeats from one or
more BAM files")
    parser.add_argument("--bam-list", '-l', required=True, help="Text file with one BAM path
per line")
    parser.add_argument("--output-dir", '-o', default="output_short_reads", help="Directory
where output files will be written")
```

```
    parser.add_argument(
        "--summary-only", '-s',
        nargs="?",
        const=True,
        default=True,
        type=str2bool,
        help="If true, writes one combined summary file; if false, writes extra per-sample
files",
    )
    return parser.parse_args()


def run_analysis_for_bam(bam_path, output_dir, summary_only):
    sample_name = extract_sample_name(bam_path)
    safe_sample_name = "".join(ch if ch.isalnum() or ch in "._-" else "_" for ch in
sample_name)

    os.makedirs(output_dir, exist_ok=True)

    total_reads = 0
    anchor_reads = 0
    selected_reads = 0
    processed_reads = set()

    class_counter = Counter()
    length_counter = Counter()

    reads_out_handle = None
    counted_out_handle = None
    counted_out_path = None
    summary_handle = None


    summary_path = os.path.join(output_dir, "summary_counts.txt")
    summary_handle = open(summary_path, "a")

    if not summary_only:
        reads_out_path = os.path.join(output_dir, f"{safe_sample_name}_reads_sequences.txt")
        counted_out_path = os.path.join(output_dir, f"{safe_sample_name}_counted_reads.txt")
        reads_out_handle = open(reads_out_path, "w")
        counted_out_handle = open(counted_out_path, "w")

    bam = pysam.AlignmentFile(bam_path, "rb")
    for read in bam.fetch(REGION_CHR, REGION_START, REGION_END):
        read_key = read.query_name
        if read_key in processed_reads:
            continue
        processed_reads.add(read_key)

        total_reads += 1
        seq = read.query_sequence

        if not seq:
            continue

        anchor_positions = find_anchor_positions(seq, ANCHOR)
        anchor2_positions = find_anchor_positions(seq, ANCHOR2)
        if not anchor_positions and not anchor2_positions:
            continue

        anchor_reads += 1

        selected, best_len, result = process_read(seq, read.query_name, reads_out_handle,
class_counter, length_counter)
        if selected:
            selected_reads += 1
            print_line = (
                f"{read.query_name}\t"
                f"len={best_len}\t"
                f"sim={result['sim']:.3f}\t"
```

```
                f"class={result['category']}\t"
                f"ref={result['reference']}"
            )
            if counted_out_handle is not None:
                counted_out_handle.write(print_line + "\n")

    bam.close()

    if reads_out_handle is not None:
        reads_out_handle.close()

    summary_lines = [
        "==============================",
        f"SUMMARY: {bam_path}",
        "",
        f"Total Reads:      {total_reads}",
        f"Anchor Reads:          {anchor_reads}",
        f"Selected Reads:      {selected_reads}",
        "",
        "Classification:",
    ]
    for key in sorted(class_counter):
        summary_lines.append(f"{key:10s} -> {class_counter[key]}")
    summary_lines.append("==============================")

    summary_handle.write("\n".join(summary_lines) + "\n\n")
    summary_handle.close()
    if not summary_only:
        with open(counted_out_path, "a") as output_handle:
            output_handle.write("\n".join(summary_lines) + "\n")
        if counted_out_handle is not None:
            counted_out_handle.close()

    return summary_lines


def main():
    args = parse_args()
    with open(args.bam_list, "r") as handle:
        bam_paths = [line.strip() for line in handle if line.strip()]

    if not bam_paths:
        raise ValueError("No BAM paths were found in the input list")

    for bam_path in bam_paths:
        glob.glob(bam_path)  # Validate the path
        if not os.path.isfile(bam_path):
            print(f"BAM file not found: {bam_path}. Skipped")
        else:
            run_analysis_for_bam(bam_path, args.output_dir, args.summary_only)


if __name__ == "__main__":
    main()
```

S3: Long-read MUC1 analysis scripts and inputs

This appendix includes the main analysis script, the configuration file, and a helper filtering script.

script_MUC1_python.py

File path: script_MUC1_python.py

```
import argparse
import pysam
from collections import Counter
from difflib import SequenceMatcher
import os
import pandas as pd
import json

def get_arguments():
    """
    Function that parse arguments given by the user, returning a dictionary
    that contains all the values
    """
    parser = argparse.ArgumentParser()
    parser.add_argument('--samples', '-s', nargs='+', required=False, help="List of sample
names")
    parser.add_argument('--base-dir', '-d', required=False, help="Base directory containing
sample BAM subfolders")
    parser.add_argument('--bam-path', help="Path to a BAM file to process directly")
    parser.add_argument('--output-dir', help="Optional output directory for generated result
files")
    parser.add_argument('--summary-only', default=True, action='store_true', help="Only
generate summary files, not individual read outputs")
    parser.add_argument('--analyze-only', action='store_true', help="Only analyze an existing
repetition_summary.tsv")
    parser.add_argument('--tsv-path', help="Path to repetition_summary.tsv for analysis")
    parser.add_argument('--config', required=True, help="Path to config.json file")
    args = parser.parse_args()
    return args


def load_config(config_path):
    """
    Load configuration from JSON file and return the config dict.
    """

    with open(config_path, 'r') as f:
        config = json.load(f)

    # Build PATTERNS dict with uppercase keys from seq_to_check
    seq_to_check = config.get("seq_to_check", {})
    config["PATTERNS"] = {k.upper(): v for k, v in seq_to_check.items()}

    return config


def match_with_N(seq, pattern, full_seq=None, start_idx=None):
    if len(seq) != len(pattern):
        return False

    for i, (a, b) in enumerate(zip(seq, pattern)):
        if b == 'N':
            continue
        elif b == 'M':
            # Just for the last position
            if i == len(pattern) - 1:
```

```
                if a == 'T' and full_seq is not None and start_idx is not None:
                    next_pos = start_idx + i + 1
                    if next_pos < len(full_seq) and full_seq[next_pos] == 'G':
                        return False
            # If sequence isn't T-->G , true
            continue
        else:
            if a != b:
                return False
    return True

def identity(a, b):
    match_a = SequenceMatcher(None, a, b).ratio()
    match_b = sum(seq1 == seq2 for seq1, seq2 in zip(a, b)) / len(a)
    if match_a > match_b:
        return match_a
    return match_b

def similarity(a, b):
    return SequenceMatcher(None, a, b).ratio()

def refine_or_blocks(seq, g_start, g_end, min_block, config,
                        sim_threshold=0.85, head_weight=0.3, body_weight=0.7,
                        block_size=60, block_tol=3):
    head_ref = config["head_ref"]
    candidates = []

    if len(seq) < min_block:
        candidates.append({"start": g_start, "end": g_end, "type": "OR", "seq": seq})
        return candidates

    for i in range(len(seq)):
        for delta in range(-block_tol, block_tol+1):
            frag = seq[i:i+block_size+delta]
            if len(frag) < block_size - block_tol or len(frag) > block_size + block_tol:
                continue
            head_frag = frag[:len(head_ref)]
            sim1 = identity(head_frag, head_ref)
            sim2 = similarity(frag, config["ref"])
            score = sim1* head_weight + sim2* body_weight
            if score >= sim_threshold:
                candidates.append({
                    "start": i,
                    "end": i + len(frag) - 1,
                    "score": score,
                    "seq": frag
                })

    candidates.sort(key=lambda x: x["end"])

    # p(j): select best compatible candidate
    p = []
    for j, c in enumerate(candidates):
        k = j - 1
        while k >= 0 and candidates[k]["end"] + block_tol >= c["start"]:
            k -= 1
        p.append(k)

    # DP
    dp = [0]*(len(candidates)+1)
    take = [False]*(len(candidates)+1)
    for j in range(1, len(candidates)+1):
        skip = dp[j-1]
        use = dp[p[j-1]+1] + candidates[j-1]["score"]
        if use > skip:
            dp[j] = use
            take[j] = True
        else:
            dp[j] = skip
```

```
    # Rebuild OV and OR blocks
    ov_blocks = []
    j = len(candidates)
    used = set()
    while j > 0:
        if take[j]:
            c = candidates[j-1]
            ov_blocks.append({
                "start": g_start + c["start"],
                "end": g_start + c["end"],
                "type": "OV",
                "seq": c["seq"]
            })
            used.update(range(c["start"], c["end"]+1))
            j = p[j-1] + 1
        else:
            j -= 1
    ov_blocks.reverse()
    # include OR blocks
    blocks = []
    last = g_start
    for ov in ov_blocks:
        if last < ov["start"]:
            # OR before OV
            blocks.append({
                "start": last,
                "end": ov["start"]-1,
                "type": "OR",
                "seq": seq[(last-g_start):(ov["start"]-g_start)]
            })
        # OV
        blocks.append({
            "start": ov["start"],
            "end": ov["end"],
            "type": "OV",
            "seq": ov["seq"]
        })
        last = ov["end"]+1
    if last <  g_start + len(seq):
        # OR after last OV
        blocks.append({
            "start": last,
            "end": g_start + len(seq) - 1,
            "type": "OR",
            "seq": seq[(last-g_start):]
        })

    return blocks

def split_regions(bam_path, output_dir, config, summary_only=False):
    bam = pysam.AlignmentFile(bam_path, "rb")
    summary = Counter({"WT": 0, "MUT": 0, "MUT2": 0,  "MUT3": 0, "OV": 0, "OR": 0})
    repeat_table = {}

    anchor_start = config["anchor_seq"]["start"]
    anchor_end = config["anchor_seq"]["end"]
    block_size = config["block_size"]
    block_tol = config["block_tol"]
    patterns = config["PATTERNS"]
    sim_threshold = config["sim_threshold"]

    for read in bam.fetch():
        seq = read.query_sequence
        read_name = read.query_name.replace("/", "_")

        # generate individual read_files
        if not summary_only:
            output_file = os.path.join(output_dir, f"{read_name}_output.txt")
            fout = open(output_file, "w")
        else:
```

```
            fout = None

        idx_start = seq.find(anchor_start)
        or_buffer = ""
        if idx_start == -1:
            summary["OR"] += 1
            if fout:
                fout.write("\n")
        else:
            idx_start += len(anchor_start)
            idx_end = seq.find(anchor_end, idx_start)
            if idx_end == -1:
                idx_end = len(seq)
            region = seq[idx_start:idx_end]
            if len(region) < block_size - block_tol:
                summary["OR"] += 1
                if fout:
                    fout.write("\n")
            else:
                i = 0
                while i < len(region):
                    found = False

                    for label, pattern in patterns.items():
                        for delta in range(-block_tol, block_tol + 1):
                            frag = region[i:i + block_size + delta]
                            if len(frag) != len(pattern):
                                continue
                            if match_with_N(frag, pattern, full_seq=region, start_idx=i):

                                # ---- Flush OR buffer ----
                                if or_buffer:
                                    or_start = i - len(or_buffer)
                                    genomic_start = read.reference_start + idx_start +
    or_start

                                    genomic_end = genomic_start + len(or_buffer) - 1
                                    list_refined = refine_or_blocks(
                                        or_buffer,
                                        genomic_start,
                                        genomic_end,
                                        block_size - block_tol,
                                        config,
                                        block_size=block_size,
                                        block_tol=block_tol,
                                        sim_threshold=sim_threshold
                                    )
                                    for block in list_refined:
                                        if fout:
                                            fout.write(
                                                f"{block['start']}-{block['end']}\t"
                                                f"{block['seq']}\t"
                                                f"{block['type']}\t"
                                                f"{block['end']-block['start']+1}\n"
                                            )
                                        summary[block['type']] += 1
                                        if block['type'] == "OR" and len(block['seq']) < 30:
                                            continue
                                        repeat_table.setdefault(read_name,
    []).append(block['type'])
                                    or_buffer = ""

                                genomic_start = read.reference_start + idx_start + i
                                genomic_end = genomic_start + len(frag) - 1

                                if fout:
                                    fout.write(
                                        f"{genomic_start}-{genomic_end}\t"
                                        f"{frag}\t"
                                        f"{label}\t"
                                        f"{genomic_end-genomic_start+1}\n"
```

```
                                    )
                                repeat_table.setdefault(read_name, []).append(label)
                                summary[label] += 1
                                i += len(frag)
                                found = True
                                break

                        if found:
                            break

                    if found:
                        continue

                    # no pattern was found
                    or_buffer += region[i]
                    i += 1
                if or_buffer:
                    or_start = i - len(or_buffer)
                    genomic_start = read.reference_start + idx_start + or_start
                    genomic_end = genomic_start + len(or_buffer) - 1
                    list_refined = refine_or_blocks(
                            or_buffer,
                            genomic_start,
                            genomic_end,
                            block_size-block_tol,
                            config,
                            block_size=block_size,
                            block_tol=block_tol,
                            sim_threshold=sim_threshold
                        )
                    for block in list_refined:
                        if fout:
                            fout.write(f"{block['start']}-
{block['end']}\t{block['seq']}\t{block['type']}\t{block['end']-block['start']+1}\n")
                        summary[block['type']] += 1
                        if block['type'] == "OR" and len(block['seq']) < 30:
                            continue
                        repeat_table.setdefault(read_name, []).append(block['type'])
        if fout:
            fout.close()
    return summary, repeat_table



def build_repetition_table(output_dir, repeat_table):
    all_reads = sorted(
        repeat_table.keys(),
        key=lambda r: (-len(repeat_table[r]), r)
    )

    max_reps = max(len(v) for v in repeat_table.values())
    output_file = os.path.join(output_dir, "repetition_summary.tsv")
    with open(output_file, "w") as fout:
        fout.write("repetition\t" + "\t".join(all_reads) + "\n")
        for rep in range(max_reps):
            row = [str(rep + 1)]
            for read in all_reads:
                labels = repeat_table[read]
                # not enough repetitions, empty
                row.append(labels[rep] if rep < len(labels) else "")
            fout.write("\t".join(row) + "\n")



def analyze_repetitions(output_dir, config):
    tsv_path = os.path.join(output_dir, "repetition_summary.tsv")
    if not os.path.exists(tsv_path):
        print(f"File {tsv_path} not found, skip analysis.")
        return

    df = pd.read_csv(tsv_path, sep='\t')
```

```
    reads = df.columns[1:]

    # Calculate read lengths
    lengths = {read: (df[read].notna() & (df[read] != "")).sum() for read in reads}
    len_values = list(lengths.values())
    if not len_values:
        print("No data to analyze.")
        return

    max_len = config["max_len"]
    patterns = config["PATTERNS"]

    len_counter = Counter(len_values)
    # Select top 2 alleles by weight (frequency * length / max_len)
    length_weights = {length: count * length / max_len for length, count in
len_counter.items()}
    sorted_lengths = sorted(length_weights.items(), key=lambda kv: (-kv[1], -kv[0]))

    # Extract top 2 alleles with their weights and sort by length (descending)
    allele_data = sorted_lengths[:2]
    top_lengths = sorted([length for length, _ in allele_data], reverse=True) if allele_data
else []

    # Assign alleles based on sorted length
    len1 = top_lengths[0] if len(top_lengths) >= 1 else None
    len2 = top_lengths[1] if len(top_lengths) >= 2 else None
    weight1 = length_weights[len1] if len1 is not None else 0
    weight2 = length_weights[len2] if len2 is not None else 0

    # Group reads by allele length
    group1 = [read for read in reads if len1 is not None and lengths[read] == len1]
    group2 = [read for read in reads if len2 is not None and lengths[read] == len2]

    print(f" allele1 (longest): {len1 if len1 is not None else 'N/A'} ({len(group1)} reads),
allele2: {len2 if len2 is not None else 'N/A'} ({len(group2)} reads)")

    all_types = list(patterns.keys()) + ["OR", "OV"]

    summary_info = {
        "total_reads": len(reads),
        "allele1_length": len1,
        "allele1_reads": len(group1),
        "allele2_length": len2,
        "allele2_reads": len(group2),
        "allele1_count": len_counter.get(len1, 0),
        "allele2_count": len_counter.get(len2, 0),
        "allele1_weight": weight1,
        "allele2_weight": weight2,
        "outliers_allele1": 0,
        "outliers_allele2": 0,
    }

    for group_name, group_reads in [('allele1', group1), ('allele2', group2)]:
        if not group_reads:
            continue

        # Calculate consensus sequence for the allele
        consensus = []
        for pos in range(len(df)):
            col_indices = df.columns.get_indexer(group_reads)
            values = df.iloc[pos, col_indices].dropna()
            counts = Counter(values)
            consensus.append(counts.most_common(1)[0][0] if counts else '')

        # Detect outliers
        threshold = 0.3
        outliers = []
        for read in group_reads:
            discrep = 0
            total = 0
```

```
            read_idx = df.columns.get_loc(read)
            for pos in range(len(df)):
                val = df.iloc[pos, read_idx]
                if pd.notna(val) and val != "":
                    total += 1
                    if val != consensus[pos]:
                        discrep += 1
            if total > 0 and discrep / total > threshold:
                outliers.append(read)

        filtered_reads = [r for r in group_reads if r not in outliers]

        print(f"  Outliers: {len(outliers)}")
        summary_info[f"outliers_{group_name}"] = len(outliers)

        # Generate stats
        for label, reads_list in [('all', group_reads), ('filtered', filtered_reads)]:
            stats = {}
            for pos in range(len(df)):
                col_indices = df.columns.get_indexer(reads_list)
                values = df.iloc[pos, col_indices].dropna()
                counts = Counter(values)
                total = sum(counts.values())
                if total > 0:
                    stats[pos+1] = {k: v/total*100 for k, v in counts.items()}

            max_len_read = max((lengths[read] for read in reads_list), default=0)

            output_stats = os.path.join(output_dir, f"{group_name}_{label}_stats.tsv")
            with open(output_stats, 'w') as f:
                f.write("position\t" + "\t".join(all_types) + "\tMajor\n")
                for pos in range(1, max_len_read + 1):
                    row = [str(pos)]
                    pos_stats = stats.get(pos, {})
                    for pat in all_types:
                        pct = pos_stats.get(pat, 0)
                        row.append(f"{pct:.2f}%")
                    # Major
                    if pos_stats:
                        major = max(pos_stats, key=pos_stats.get)
                    else:
                        major = ""
                    row.append(major)
                    f.write("\t".join(row) + "\n")

        # Safe outliers
        if outliers:
            output_outliers = os.path.join(output_dir, f"{group_name}_outliers.txt")
            with open(output_outliers, 'w') as f:
                f.write("\n".join(outliers))

    total_outliers = summary_info.get("outliers_allele1", 0) +
summary_info.get("outliers_allele2", 0)
    summary_info["total_outliers"] = total_outliers
    summary_info["allele1_pct"] = summary_info["allele1_reads"] / summary_info["total_reads"]
* 100 if summary_info["total_reads"] else 0
    summary_info["allele2_pct"] = summary_info["allele2_reads"] / summary_info["total_reads"]
* 100 if summary_info["total_reads"] else 0
    summary_info["outliers_pct"] = total_outliers / summary_info["total_reads"] * 100 if
summary_info["total_reads"] else 0

    summary_path = os.path.join(output_dir, "repetition_analysis_summary.txt")
    with open(summary_path, 'w') as f:
        f.write(f"total_reads: {summary_info['total_reads']}\n")
        f.write(f"allele1_length: {summary_info['allele1_length']}\n")
        f.write(f"allele1_reads: {summary_info['allele1_reads']}
({summary_info['allele1_pct']:.1f}%)\n")
        f.write(f"allele1_weight: {summary_info['allele1_weight']:.2f}\n")
        f.write(f"allele2_length: {summary_info['allele2_length']}\n")
        f.write(f"allele2_reads: {summary_info['allele2_reads']}
```

```
({summary_info['allele2_pct']:.1f}%)\n")
        f.write(f"allele2_weight: {summary_info['allele2_weight']:.2f}\n")
        f.write(f"non-associated allele reads: {summary_info['total_reads'] -
summary_info['allele1_reads'] - summary_info['allele2_reads']} ({100 -
summary_info['allele1_pct'] - summary_info['allele2_pct']:.1f}%)\n")

        f.write(f"outliers_allele1: {summary_info['outliers_allele1']}\n")
        f.write(f"outliers_allele2: {summary_info['outliers_allele2']}\n")
        f.write(f"total_outliers: {summary_info['total_outliers']}
({summary_info['outliers_pct']:.1f}%)\n")

    print("Completed analysis.")


def load_table_to_df(table: dict, output_path="debug_df7.csv"):
    df = pd.DataFrame.from_dict(table, orient='index').fillna('')
    df.to_csv(output_path)
    print(f"✅ DataFrame saved: {output_path}")
    return df

def main():
    print("INFO: start execution")
    args = get_arguments()
    config = load_config(args.config if hasattr(args, 'config') and args.config else None)

    if args.analyze_only and args.tsv_path:
        output_dir = os.path.dirname(args.tsv_path)
        analyze_repetitions(output_dir, config)
        return

    if args.bam_path:
        if not os.path.exists(args.bam_path):
            print(f"ERROR: BAM file not found: {args.bam_path}")
            return

        bam_path = args.bam_path
        bam_base = os.path.splitext(os.path.basename(bam_path))[0]
        bam_dir = os.path.dirname(bam_path) or '.'
        if args.output_dir:
            output_dir = args.output_dir
        else:
            output_dir = os.path.join(
                bam_dir,
                f"{bam_base}_muc1_summary_results" if args.summary_only else
f"{bam_base}_muc1_all_reads"
            )

        os.makedirs(output_dir, exist_ok=True)
        print(f"\nProcessing BAM file: {bam_path}")

        summary, repeat_table = split_regions(bam_path, output_dir, config,
summary_only=args.summary_only)
        build_repetition_table(output_dir, repeat_table)
        analyze_repetitions(output_dir, config)

        print(f"\n✅ BAM file processed: {bam_path}")
        print(f"   - Summary counts (including outliers): {dict(summary)}")
        print(f"   - Total reads: {len(repeat_table)}")
        print(f"   - Output directory: {output_dir}")
        return

    if not args.samples or not args.base_dir:
        print("ERROR: For complete processing, the parameters --samples and --base-dir are
required if --bam-path is not provided.")
        return

    for sample in args.samples:
        sample_dir = os.path.join(args.base_dir, sample)
        bam_path = os.path.join(sample_dir, f"{sample}_filtered.bam")
```

```
        output_dir = os.path.join(
            sample_dir,
            f"{sample}_muc1_summary_results2" if args.summary_only else
f"{sample}_muc1_reads_outputs_def_version"
        )

        if not os.path.exists(sample_dir):
            print(f"ERROR: Sample directory not found: {sample_dir}")
            continue
        if not os.path.exists(bam_path):
            print(f"ERROR: BAM file not found: {bam_path}")
            continue
        os.makedirs(output_dir, exist_ok=True)
        print(f"\nProcessing sample: {sample}")

        summary, repeat_table = split_regions(bam_path, output_dir, config,
summary_only=args.summary_only)
        build_repetition_table(output_dir, repeat_table)
        analyze_repetitions(output_dir, config)

        print(f"\n✅ Sample processed: {sample}")
        print(f"   - Summary counts (including outliers): {dict(summary)}")
        print(f"   - Total reads: {len(repeat_table)}")
        print(f"   - Output directory: {output_dir}")


if __name__ == "__main__":
    main()
```

config.json

File path: inputs/config.json

```
{
    "bam_path": "/",
    "output_dir": "/",
    "head_ref": "TGGGGGGGCGGT",
    "ref": "TGGGGGGGCGGTGGAGCCCGGGGCCGGCCTGGTGTCCGGGGCCGAGGTGACACCGTGGGC",
    "seq_to_check":{
        "wt": "TGGGGGGGCGGTNNNNNNNNNNNNNNNNNNNNNNNNNNNNNNNNNNNNNNNNNNNNNNNM",
        "mut": "TGGGGGGGGCGGTNNNNNNNNNNNNNNNNNNNNNNNNNNNNNNNNNNNNNNNNNNNNNNNM",
        "mut2": "TTGGGGGGGCGGTNNNNNNNNNNNNNNNNNNNNNNNNNNNNNNNNNNNNNNNNNNNNNNNM",
        "mut3": "TTGGGGGGCGGTNNNNNNNNNNNNNNNNNNNNNNNNNNNNNNNNNNNNNNNNNNNNNNNM",
        "mut4": "TGGGGGGCGGTNNNNNNNNNNNNNNNNNNNNNNNNNNNNNNNNNNNNNNNNNNNNNNNM"
    },
    "anchor_seq":{
        "start": "TGAGCCTGATGCAGAGCCTGAGGCCGAGGTGACATTGTGGACTGGAGGGG",
        "end": "GCGGTGGAGCCCGGGGCTGGCTTGTTGTCCGGGGCTGAGGTGACATCGTG"
    },
    "block_size": 60,
    "block_tol": 2,
    "sim_threshold": 0.85,
    "max_len": 200
}
```

filter_reads.sh

File path: scripts/filter_reads.sh

```
#!/bin/bash

INPUT_BAM="$1"
SAMPLE="$2"

if [ "$#" -ne 2 ]; then
    echo "Uso: $0 <input.bam> <sample>"
    exit 1
fi

OUTPUT_DIR="samples/${SAMPLE}"
OUTPUT_BAM="${OUTPUT_DIR}/${SAMPLE}_filtered.bam"

mkdir -p "$OUTPUT_DIR"

samtools view -h "$INPUT_BAM" \
| awk '/^@/ || ($0 ~ /TGAGCCTGATGCAGAGCCTGAGGCCGAGGTGACATTGTGGACTGGAGGGG/ && $0 ~
/GCGGTGGAGCCCGGGGCTGGCTTGTTGTCCGGGGCTGAGGTGACATCGTG/)' \
| samtools view -b -o "$OUTPUT_BAM"

samtools index "$OUTPUT_BAM"

echo "BAM generated: $OUTPUT_BAM"
```
